# Proteomic Analysis of *Ketogulonicigenium vulgare* under Glutathione Reveals High Demand for Thiamin Transport and Antioxidant Protection

**DOI:** 10.1371/journal.pone.0032156

**Published:** 2012-02-22

**Authors:** Qian Ma, Weiwen Zhang, Lu Zhang, Bin Qiao, Chensong Pan, Hong Yi, Lili Wang, Ying-jin Yuan

**Affiliations:** 1 Key Laboratory of Systems Bioengineering, Ministry of Education and Department of Pharmaceutical Engineering, School of Chemical Engineering and Technology, Tianjin University, Tianjin, People's Republic of China; 2 Beijing Office, Bruker Daltonics Inc., Beijing, People's Republic of China; 3 College of Biology Science and Engineering, Hebei University of Science and Technology, Shijiazhuang, Hebei, People's Republic of China; University of South Florida College of Medicine, United States of America

## Abstract

*Ketogulonicigenium vulgare*, though grows poorly when mono-cultured, has been widely used in the industrial production of the precursor of vitamin C with the coculture of *Bacillus megaterium*. Various efforts have been made to clarify the synergic pattern of this artificial microbial community and to improve the growth and production ability of *K. vulgare*, but there is still no sound explanation. In previous research, we found that the addition of reduced glutathione into *K. vulgare* monoculture could significantly improve its growth and productivity. By performing SEM and TEM, we observed that after adding GSH into *K. vulgare* monoculture, cells became about 4–6 folds elongated, and formed intracytoplasmic membranes (ICM). To explore the molecular mechanism and provide insights into the investigation of the synergic pattern of the co-culture system, we conducted a comparative iTRAQ-2-D-LC-MS/MS-based proteomic analysis of *K. vulgare* grown under reduced glutathione. Principal component analysis of proteomic data showed that after the addition of glutathione, proteins for thiamin/thiamin pyrophosphate (TPP) transport, glutathione transport and the maintenance of membrane integrity, together with several membrane-bound dehydrogenases had significant up-regulation. Besides, several proteins participating in the pentose phosphate pathway and tricarboxylic acid cycle were also up-regulated. Additionally, proteins combating intracellular reactive oxygen species were also up-regulated, which similarly occurred in *K. vulgare* when the co-cultured *B. megaterium* cells lysed from our former research results. This study reveals the demand for transmembrane transport of substrates, especially thiamin, and the demand for antioxidant protection of *K. vulgare*.

## Introduction

L-Ascorbic acid (L-AA), also known as vitamin C, has been widely used in pharmaceutical, food and cosmetic industries mainly for its antioxidant property [1], [2]. Seven-step Reichstein method has been employed to produce vitamin C since 1934 [3]. However, high energy cost and chemical pollution have made this chemical-based method less attractive. In China, a two-step microbial fermentation process was developed [4] and has been successfully used for industrial production of vitamin C [5]. The fermentation process consists of one step of conversion from D-sorbitol to L-sorbose by *Gluconobacter oxydans* [6], followed by another step of converting L-sorbose to 2-keto-gulonic acid (2-KGA), the precursor of vitamin C, by a mixed culture system of *Ketogulonicigenium vulgare* and *Bacillus megaterium* [6], [7], [8]. In the mixed culture, the 2-KGA producing bacteria *K. vulgare* which grows poorly when cultivated alone even on rich media [9], can grow better and carry out the conversion with the coculture of *B. megaterium* with high efficiency [8]. In spite of obvious advantages over monoculture of *K. vulgare*, the mixed culture has relatively higher cost for fermentation and contamination issue caused by the *B. megaterium* spore [10].

Previously, Leduc *et al* added 100 µg/L of reduced glutathione (GSH) into the *K. vulgare* LMP P-20356 monoculture [11], but no growth improvement of *K. vulgare* was observed. However, we have recently found that the addition of 1 g/L GSH into the *K. vulgare* monoculture could considerably enhance cell growth and improve 2-KGA production [12]. The discrepancy of our result and that of Leduc's may be due to different concentrations of GSH used, and could also be due to the species differences between two studies. In addition, we also found that *K. vulgare* cells elongated after GSH treatment. *K. vulgare*, formerly classified in *Gluconobacter oxydans* [13], belongs to the α-Proteobacteria. It was reported that *G. oxydans* when confronted with certain stresses could form intracytoplasmic membranes (ICM) at the end of exponential phase [14]. Accompanying the ICM formation, cells became longer and the activities of several membrane-bound dehydrogenases including glycerol [14], sorbitol and other polyol dehydrogenases [15] were improved significantly. Improved 2-KGA production suggested probable enhanced activities of two membrane-bound dehydrogenases (sorbose dehydrogenase and sorbosone dehydrogenase) in *K. vulgare* responsible for the conversion of L-sorbose to 2-KGA. Thus, it could be speculated that similar ICM formation might also occurred in *K. vulgare* grown under GSH treatment. After our transmission electron microscopy observation, this speculation was confirmed. Proteomic analysis has been successfully applied in our laboratory to study cellular responses of *Saccharomyces cerevisiae* to external stimuli [16], [17]. Considering its accuracy in quantification and relative lower protein amount requirement, iTRAQ (isotopic Tags for Relative and Absolute Quantification)-coupled 2-D LC-MS/MS technique was used in this study to identify and quantify differential proteomes in *K. vulgare* cells grown with and without GSH, with the aims to reveal the molecular mechanism of GSH's enhancing effects, to study the similarities and differences between the mechanisms of GSH and *Bacillus megaterium* on *K. vulgare* and further in order to provide insights into the study of the synergism of the co-culture system, and ultimately to find possible targets of gene modification in *K. vulgare* for further improving cell growth and 2-KGA production. The results showed that significant changes in the proteome of *K. vulgare* occurred at 15 h after GSH addition. After principal component analysis (PCA), a series of membrane related proteins involved in thiamin/thiamin pyrophosphate (TPP) transport, oligopeptide transport, outer membrane integrity maintenance, and L-sorbose metabolism were found to contribute the most to the distinguishment of the GSH addition group at 15 h. Several proteins in pentose phosphate pathway (PPP) and tricarboxylic acid (TCA) cycle also showed up-regulation. Besides, some proteins combating against intracellular reactive oxygen species (ROS) were also up-regulated, which resembled our former research results when the *B. megaterium* cells lysed in the mix-culture system [18], suggesting a reducing environment should be important for the growth and 2-KGA production of *K. vulgare*, and this demand could be satisfied either by the addition of *B. megaterium*, or by the addition of reduced GSH.

## Results and Discussion

### Physiological Profile and Morphology of *K. vulgare* Treated with and without GSH

**Figure 1:**
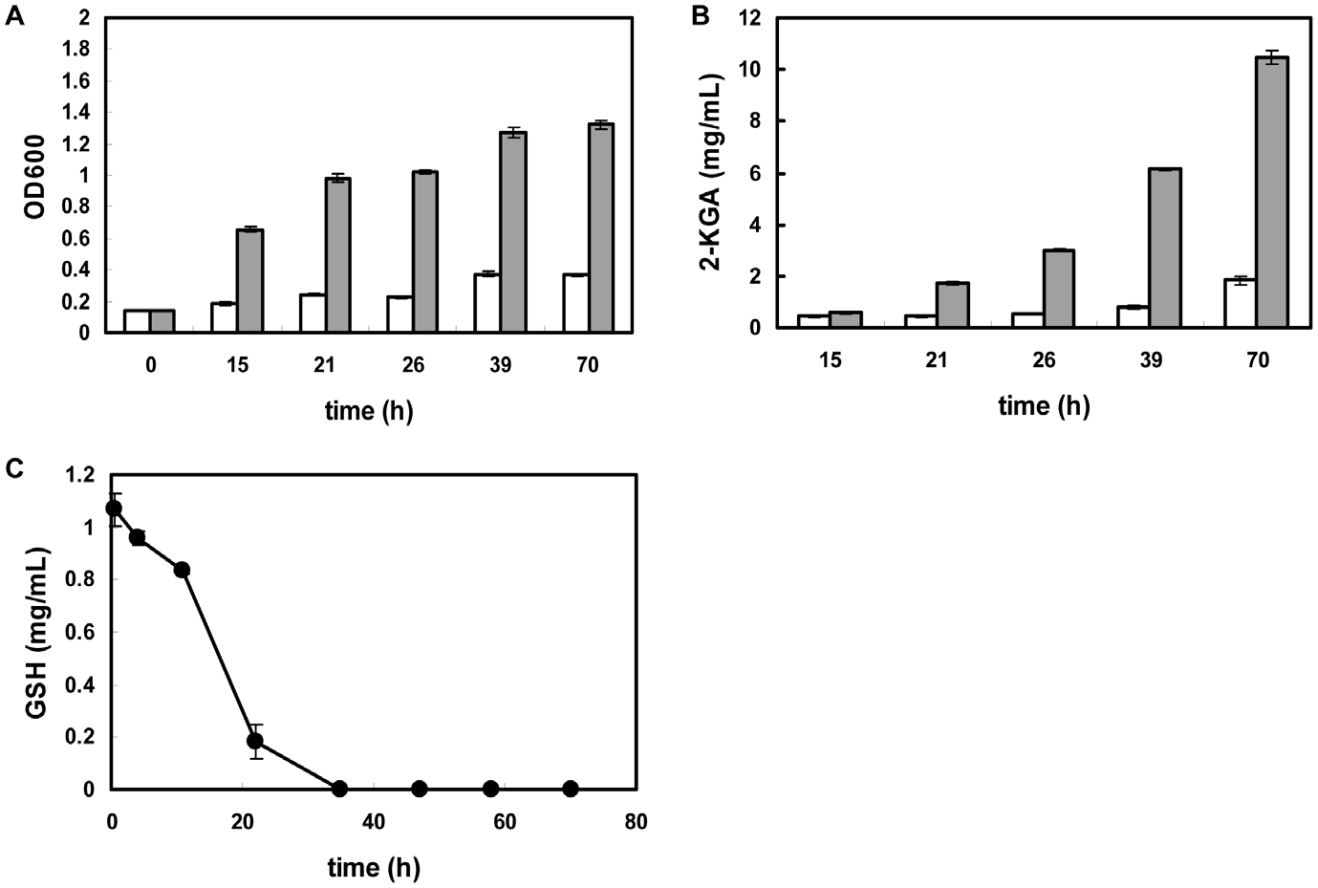
Growth, 2-KGA production of *K. vulgare* and extracellular GSH content: (A) Optical density of *K. vulgare*; (B) 2-KGA production of *K. vulgare*; (C) extracellular GSH content. (gray column represents the *K. vulgare* with GSH addition, white column represents the *K. vulgare* control, circle filled with black represents extracellular GSH)

**Figure 2:**
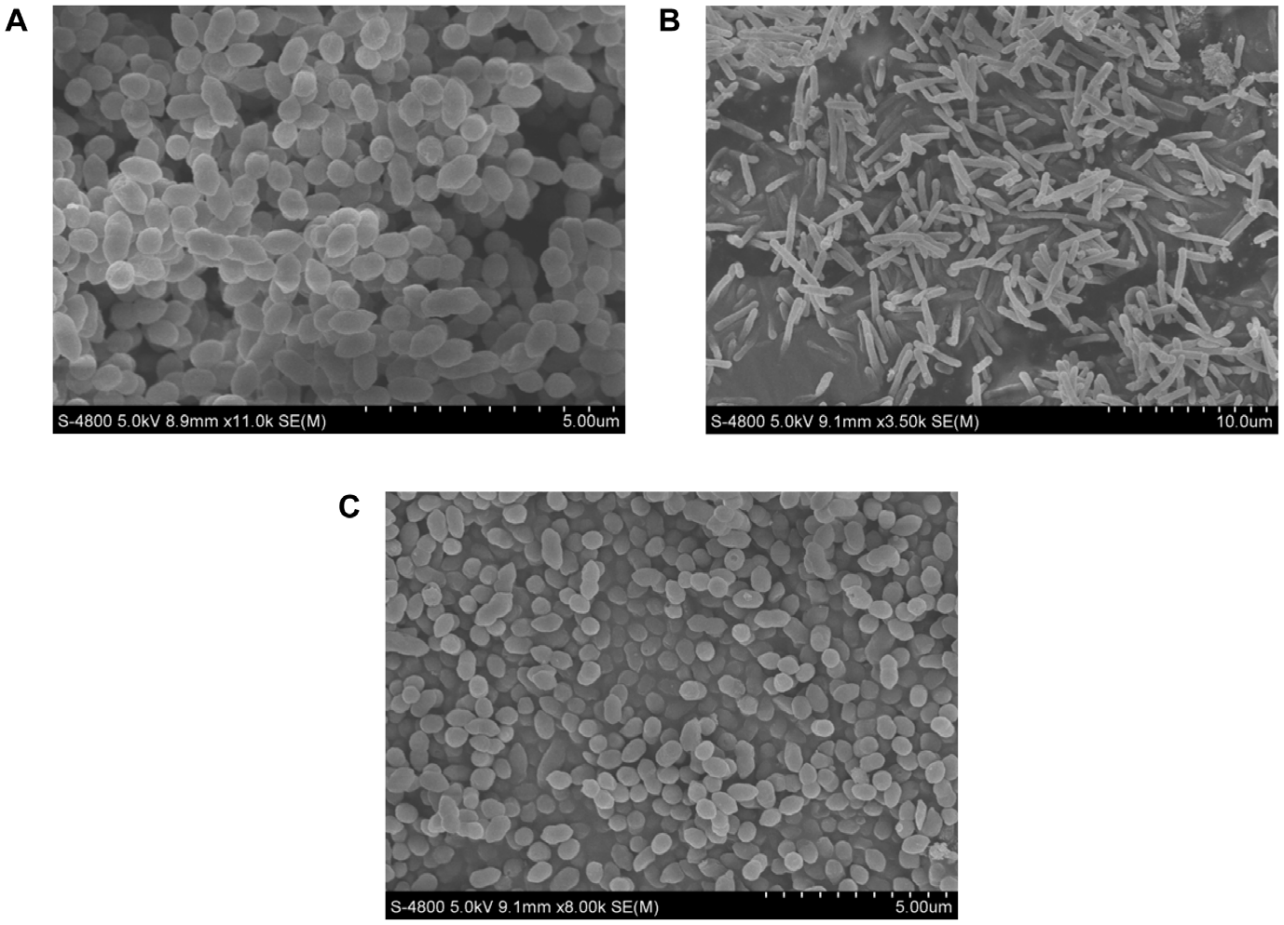
Scanning electron microscope observation of *K. vulgare* morphology: (A) *K. vulgare* without GSH addition at 24 h of fermentation; (B) *K. vulgare* with GSH addition at 24 h of fermentation; (C) *K. vulgare* transferred to agar plate after 24 h of fermentation with GSH.

A comparative physiological study of *K. vulgare* with and without GSH addition was used to determine the influence of GSH on *K. vulgare* (Figure 1). Compared with the control group, *K. vulgare* with 1 g/L GSH addition could achieve 3.6-fold of growth and 5.6-fold of 2-KGA production at 70 h of the fermentation. The extracellular GSH content was shown in Figure 1C, suggesting that GSH in the environment was completely depleted after 36 h. The cells were harvested at 1 h and 15 h after adding GSH, which represented the beginning and middle phases of GSH depletion, respectively. Scanning electron microscopy (SEM) was used to determine the morphology change of *K. vulgare* (Figure 2), and after measuring cell size in 20 replicates, we obtained the following results: the size of the *K. vulgare* cell without GSH addition was about (0.6±0.2)×(0.5±0.1) µm (Figure 2A), and at 24 h after GSH addition, the cell size became (2.4±0.7)×(0.6±0.1) µm (Figure 2B). Interestingly, when the elongated cells resulting from GSH treatment were transferred to agar plate, the cells were able to change back to their original size (Figure 2C), indicating the variation in morphology was reversible, largely depending on the environmental conditions.

**Figure 3:**
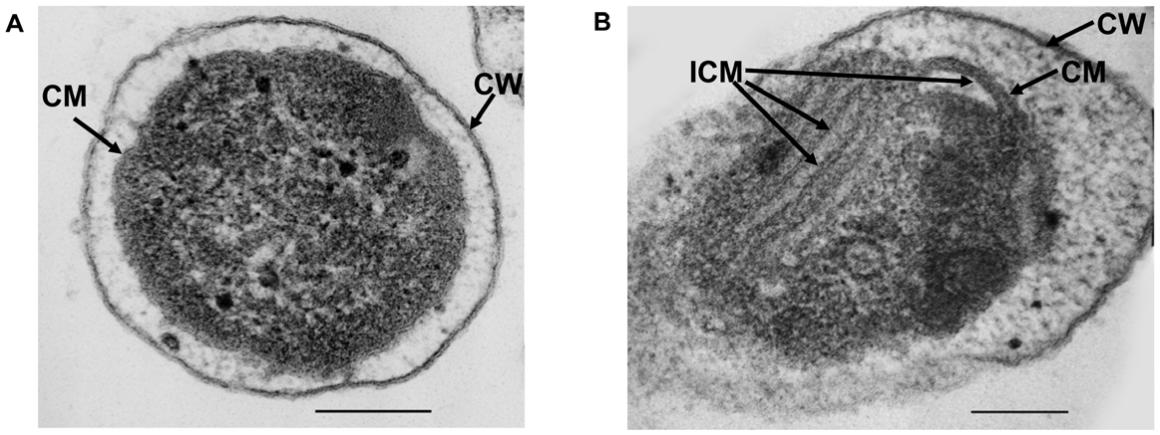
Transmission electron microscope observation of *K. vulgare* morphology: (A) *K. vulgare* without GSH addition at 24 h of fermentation; (B) *K. vulgare* with GSH addition at 24 h of fermentation. (the black bar represents a length of 0.2 µm; CW: cell wall; CM: cell membrane; ICM: intracytoplasmic membrane)

Similar change in cell morphology also occurred to *G. oxydans* as described previously [14], [15], [19]. It was reported that among the non-photosynthetic gram-negative prokaryotes, complex ICM were formed most notably in four physiological groups: *Azotobacter*, *Gluconobacter*, the obligate methane and methanol oxidizers, and the ammonium of nitrite oxidizers [15]. Studies with *G. oxydans* showed that viable cells became two to four times longer as a result of the differentiation at the end of exponential growth by forming dense regions containing accumulations of ICM and ribosomes at the end of each cell [14]. It was also revealed that several membrane-bound dehydrogenases for glycerol, sorbitol and other polyols acquired improved activities probably as the ICM were extensions of plasma membranes [14]. In our study, the cells had a 4–6 folds of cell elongation and 5.6 folds of 2-KGA producion by the membrane-bound sorbose/sorbosone dehydrogenase. Considering the homology between *K. vulgare* and *G. oxydans*, similar cell differentiation and ICM forming under GSH stimulation are quite possible. In order to confirm our speculation, transmission electron microscopy (TEM) observation was conducted, and the resluts were shown in Figure 3. From Figure 3B, we can see that after adding GSH into *K. vulgare* monoculture, the cells formed ICM as we conjectured.

### Protein Function Analysis

**Figure 4:**
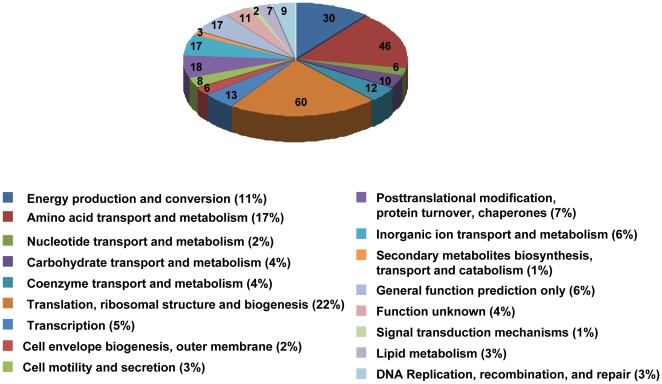
Protein function classifications of *K. vulgare* by COGnitor. Identified protein number for each category was depicted on the pie, and their relative proportions were expressed in percentage values following each ontology term description.

Proteomic analysis was employed to seek further clues on GSH's effects. A total of 319 proteins were identified from three biological replicates by 2-D nano-LC-MS/MS, and among them 265 proteins were quantified. The quantified proteins were listed in Table S1. Phylogenetic classification of the identified proteins was performed by searching the Clusters of Orthologous Groups of proteins (http://www.ncbi.nlm.nih.gov/COG) by COGnitor. As shown in Figure 4, the three major protein categories were proteins for translation, ribosomal structure and biogenesis (22%), followed by amino acid transport and metabolism (17%), and energy production and conversion (11%). Proteins involved in posttranslational modification, protein turnover, chaperones, transcription and other 13 categories of functions were also identified. In our previous study on the metabolic cooperation of *K. vulgare* and *B. megaterium* by spatially cultivating them on a solid agar plate [20], we have found that *K. vulgare* had the ability of secreting amino acids to the environment, suggesting *K. vulgare* itself may have a high-efficiency amino acid transport system. In our former proteomic analysis of the mix-culture system [18], the proportion of proteins for amino acid transport and metabolism in *K. vulgare* accounted for 22%, higher than that in the monoculture with GSH, suggesting in the mix culture *B. megaterium* might helped amino acid transport of *K. vulgare* to a larger extent than GSH. Proteins with just 4% proportion for carbohydrate transport and metabolism was probably correlated with the deficient TCA cycle and poor PPP in *K. vulgare*, while in the mix-culture system, the proportion increased to 8% [18], revealing the significant assistance of *B. megaterium* on the central carbon metabolism of *K. vulgare*.

Among the 41 proteins participating in translation, ribosomal structure and biogenesis, 31 were up-regulated for over 2 folds in *K. vulgare* with GSH addition at 15 h, compared with the control group, consistent with previous report that the differentiation of *G. oxydans* caused the accumulation of ribosomes at the poles of each cell [14]. With the increase in expression of ribosome proteins, more mRNA could be translated into amino acids, then polypeptide chain, and ultimately protein.

### PCA Analysis of the *K. vulgare* Proteins

**Figure 5:**
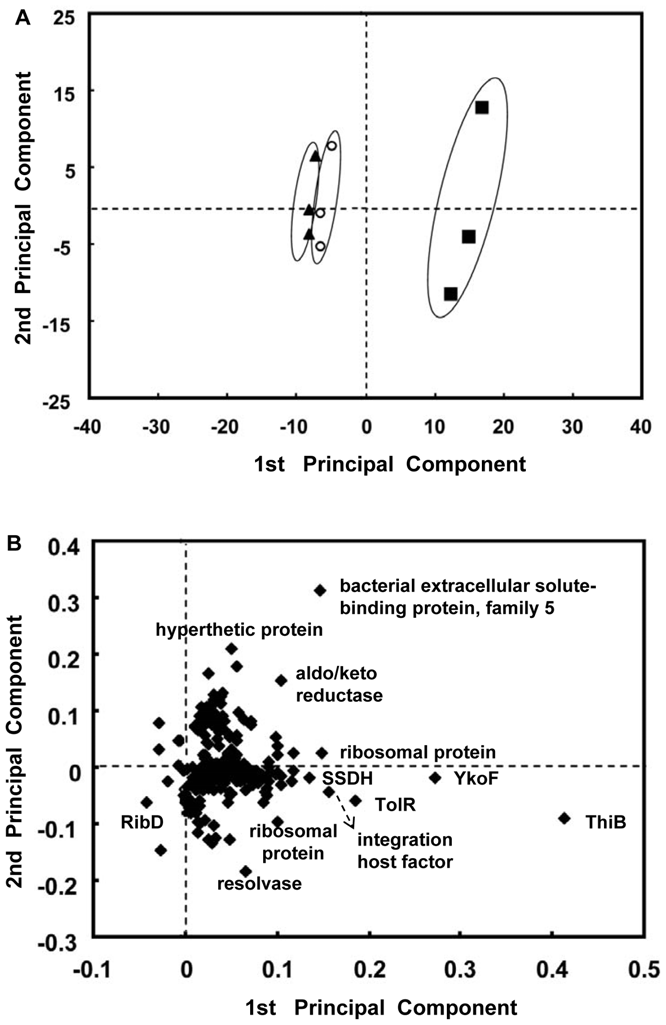
Principal component analysis of protein data: (A) scores plot of the samples (triangle filled with black represents the 115/114 group (GSH-1 h/Kv-1 h), circle filled with white represents the 116/114 group (Kv-15 h/Kv-1 h), square filled with black represents the 117/114 group (GSH-15 h/Kv-1 h)); (B) loading plot of principal component 1 vs. principal component 2.

In this study, we applied PCA to the quantified proteins, and the result was shown in Figure 5. In the score plot in Figure 5A, the datasets from 115/114, 116/114, and 117/114, which represented GSH-1 h, Kv-15 h and GSH-15 h compared with Kv-1 h, respectively, could be discriminated as circled by the three ellipses. Especially, the 117/114 dataset was conspicuously away from the other two datasets, which indicated significant protein variations had occurred in the GSH treated *K. vulgare* at 15 h. While the 115/114 and 116/114 groups didn't show much distinction, suggesting that *K. vulgare* didn't have much variation during its monoculture process, and yet no significant changes occurred in protein level at 1 h after the addition of GSH. The loading plot in Figure 5B showed that thiamin/thiamin pyrophosphate-binding protein (ThiB), YkoF-related family protein, bacterial extracellular solute-binding proteins, family 5 Middle family protein, protein TolR, sorbose/sorbosone dehydrogenase (SSDH), resolvase, ribosomal protein, integration host factor were important proteins for the discrimination of the 117/114 group from the other two groups.

**Figure 6:**
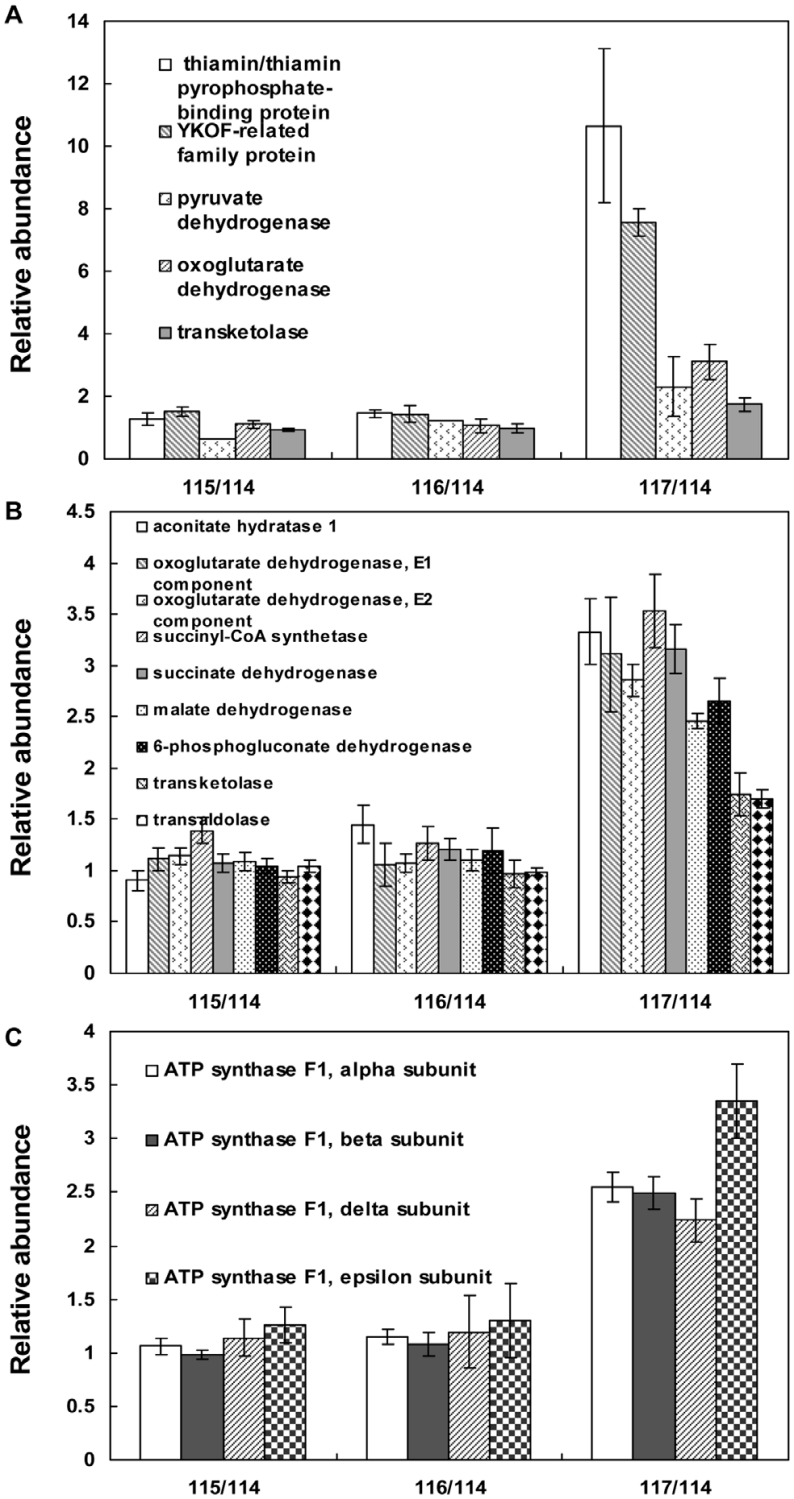
Profiles of proteins involved in TPP transport and carbohydrate metabolism: (A) TPP transport related proteins and proteins depending on TPP as coenzyme; (B) proteins identified and quantified in TCA cycle and PPP; (C) ATP synthase subunits.

ThiB, which belongs to the ThiBPQ transport complex for thiamin and TPP, is used as a marker of the complex [21]. Besides, the YkoF protein is also involved in thiamin transport [22]. These two proteins were the most up-regulated proteins in *K. vulgare* in response to GSH at 15 h compared with the control (thiB, 7.4 folds; YkoF, 5.3 folds), and their relative abundances were shown in Figure 6A, suggesting these two proteins possibly have close relationship with the GSH stimulation. The similarities of these two proteins in their functions led us to examine the function of thiamin (vitamin B_1_). After its uptake by cells, thiamin undergoes pyrophosphorylation yielding thiamin pyrophosphate (TPP), which is the active substance [23]. TPP participates in carbohydrate metabolism as an important coenzyme for pyruvate dehydrogenase, alpha-ketoglutarate dehydrogenase and transketolase [24], and thus is important to TCA cycle and PPP by affecting their key enzymes. The relative abundances of these three enzymes were also shown in Figure 6A, which all showed up-regulation in *K. vulgare* grown with GSH at 15 h. *K. vulgare* was reported to have a deficient TCA cycle, and mainly depended on PPP to carry out central carbon metabolism [25]. While in *K. vulgare* with GSH addition, five important enzymes in TCA cycle got more than 2-fold up-regulation, and three enzymes in PPP got more than 1.7-fold up-regulation (Figure 6B). In addition, increased ATP synthesis could be observed from Figure 6C, in which four subunits of ATP synthase were up-regulated to about 2–3 folds at 15 h under the stimulation of GSH. These results suggested that the addition of GSH might facilitate the thiamin/TPP transport and then improve the activities of important enzymes in TCA cycle and PPP. In this way, more ATP could be generated and in return be used to supply energy for GSH transport itself and also thiamin/TPP transport, forming a positive circulation.

**Figure 7:**
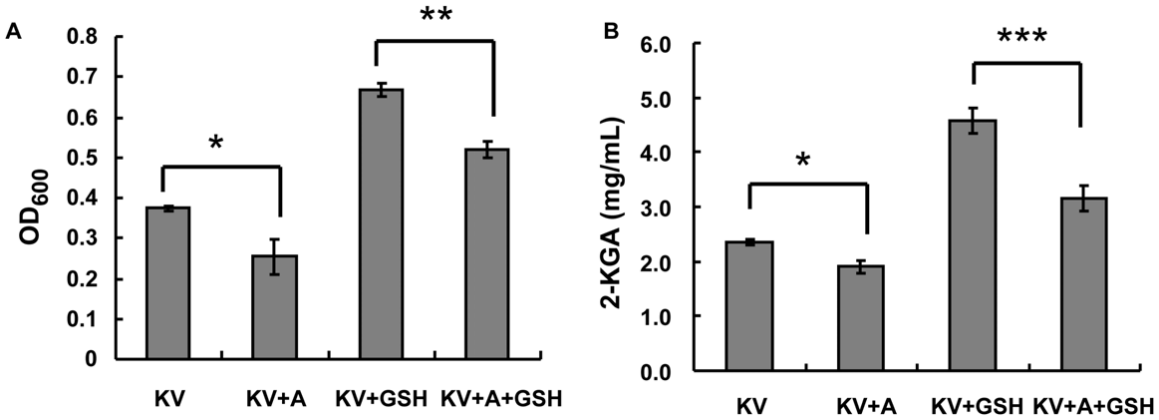
Growth and 2-KGA production of *K. vulgare* under amprolium and glutathione treatment at 24 h: (A) Optical density of *K. vulgare*; (B) 2-KGA production of *K. vulgare* (KV: *K. vulgare*; KV+A: *K. vulgare* with amprolium; KV+GSH: *K. vulgare* with GSH; KV+A+GSH: *K. vulgare* with both amprolium and GSH.)

In order to validate the conjecture of the importance of thiamin transport to *K. vulgare*, we performed a negative validation by adding inhibitor of thiamin transport-amprolium, which competed with thiamin in being transported as an analogue [26], [27]. It had been used in *E. coli* as a thiamin analogue to explore the molecular recognition characteristics of the TPP riboswitch [27]. In our study, a final concentration of 1 mg/mL of amprolium was added to *K. vulgare* monoculture and *K. vulgare* with GSH addition, respectively. The growth and 2-KGA production of *K. vulgare* under different treatments were shown in Figure 7, from which we could see that after a culture of 24 h, amprolium slightly lowered the growth and 2-KGA production of mono-cultured *K. vulgare*, while in *K. vulgare* with both amprolium and GSH, the growth and 2-KGA produciton decreased to a considerable larger extent. These results suggested that thiamin played an essential role in the growth and 2-KGA production of *K. vulgare*, and that GSH could enhance the transport process regardless of thiamin or its analogue.

Considering the importance of thiamin transport process, there is a possibility that adding thiamin into the *K. vulgare* monoculture might improve cell growth. In our research, we added thiamin with a final concentration of 0.2 mg/mL. While from Figure S1, we didn't see much difference in growth and 2-KGA production of *K. vulgare* after adding thiamin. This phenomenon was probably due to the fact that active transport process was not only substrate restricted, but also closely related with indispensable trasnporter and energy support. As a poor growing bacterium, *K. vulgare* has defects in both upstream protein synthesis and downstream metabolism including energy production. Thus, in order to improve its gowth and production ability, a global regulation should take place. The response of *K. vulgare* to glutathione was such a global regulation.

**Figure 8:**
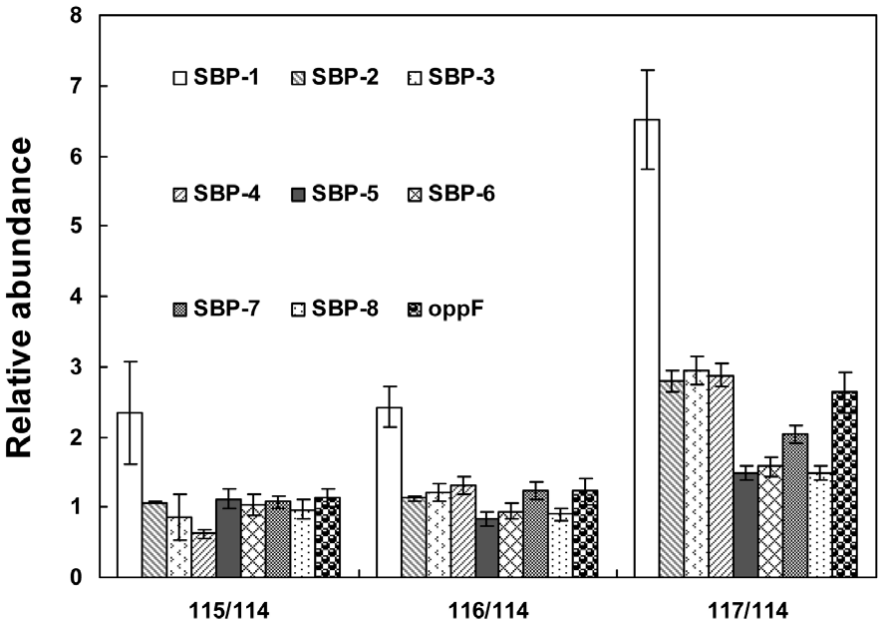
Profiles of oligopeptide transport related proteins: “SBP” represents bacterial extracellular solute-binding protein, family5.

**Figure 9:**
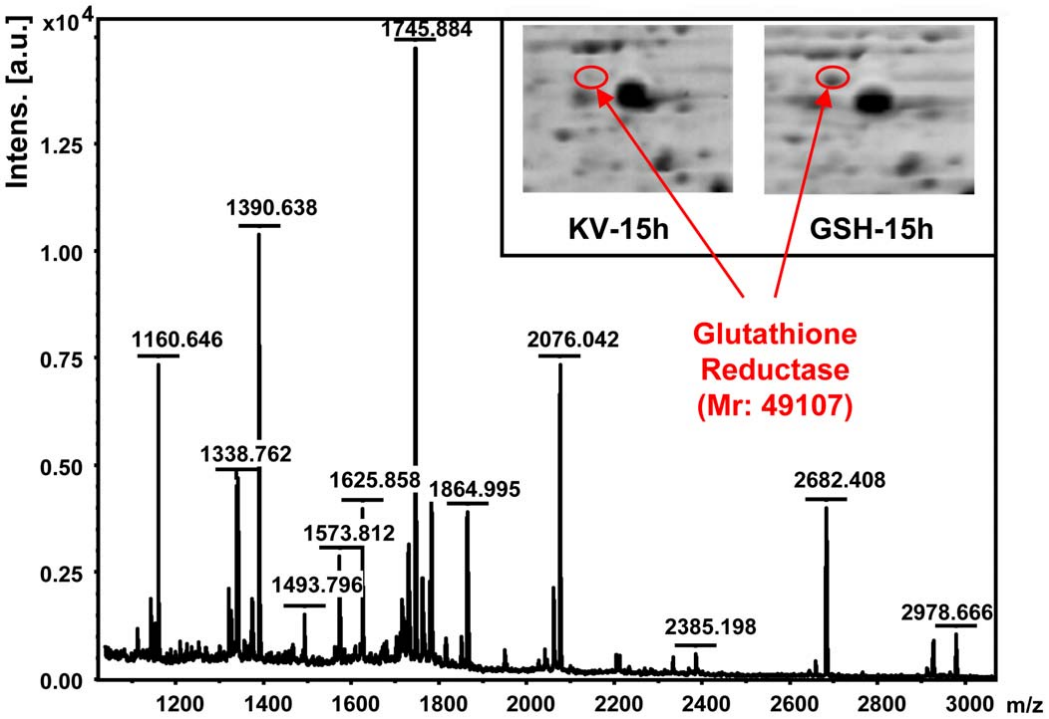
Peptide mass fingerprint of glutathione reductase and its expression in *K. vulgare* grown with and without GSH.

Bacterial extracellular solute-binding proteins, participating in active transport of solutes across the cytoplasmic membrane, generally correlated with the nature of the solute bound, are grouped into eight families of clusters. The family 5 mainly consists of periplasmic oligopeptide-binding protein (oppA), dipeptide-binding protein, nickel-binding proteins, and heme-binding lipoprotein. It was reported in *Bacillus subtilis* and *Lactococcus lactis* that the oligopeptide transport system (Opp) was encoded by the gene *oppDFBCA* [28], [29]. This transport system typically transports peptides of 3 to 5 amino acids, independently of peptide sequence [28]. According to genome sequencing results, the Opp system is also present in *K. vulgare*. GSH, a tripeptide in the form of L-GluCysGly, could be imported into the cell through the Opp system. In this study, we have identified several bacterial extracellular solute-binding proteins, family 5 middle family protein, and also the protein OppF, which links transport to the hydrolysis of ATP as an ATP binding protein [28]. The profiles for these proteins were shown in Figure 8. Among which SBP-1 had a 2.4-fold up-regulation compared with the control (115/114) at 1 h after GSH addition, which revealed that the oligopeptide transport in *K. vulgare* started to be enhanced shortly after the additon of GSH. In our former proteomic study of the co-culture system, similar up-regulation of bacterial extracellular solute-binding proteins, family 5 middle family protein also occurred when the cell lysis of *B. megaterium* took place during the mix-culture fermentation, which suggests the cell lysis of *B. megaterium* also stimulated the uptake of oligopeptide by *K. vulgare*. Considering the conversion between reduced GSH and GSSG, glutathione reductase (GR) is important so as to keep a relative higher GSH/GSSG ratio. Not only the transport of GSH, but also the GR abundance should play important role in the function of GSH inside the cells. However, perhaps due to the separation limitation, we didn't find this protein by LC-MS detection. Then in order to find this protein, we tried 2-D gel separation for its good separation properties. After theoretical molecular weight/PI calculation based location, and further peptide mass fingerprint identification, we found that in the control, the spot for GR was hardly visible, while in the GSH treated *K. vulgare*, GR had a certain expression, as shown in Figure 9. In this way, we suppose GSH could positively function inside the cells. Protein TolR is a part of the Tol/Pal system which forms a five-member, membrane-spanning, multiprotein complex. The complex has been found conserved in gram-negative bacteria and involves the integrity of the outer membrane [30], [31], [32]. In our study, the abundance of TolR had a 3.4-fold up-regulation after the GSH addition at 15 h. The up-regulation of oligopeptide transport proteins and protein TolR, together with ThiB and YkoF, suggested GSH brought significant effects on cell membranes of *K. vulgare*.

Previous studies revealed that the conversion of L-sorbose to 2-KGA comprised two successive reactions: one is the oxidation of L-sorbose into L-sorbosone by sorbose dehydrogenase [33], and the other is the subsequent oxidation of L-sorbosone into 2-KGA by sorbosone dehydrogenase [13]. Both of these two dehydrogenases are membrane bounded and pyrroloquinoline quinone (PQQ)-dependent [34]. In our investigation, these two enzymes in the form of sorbose/sorbosone dehydrogenase, had an up-regulation of 3.6 folds in *K. vulgare* at 15 h after GSH addition. This phenomenon together with cell elongation, resembled the case of enhanced sorbitol dehydrogenase activity and cell elongation in *G. oxydans* as a result of the ICM formation [15]. Considering the above similarities, it is quite probable that ICM forming occurred in *K. vulgare* under GSH treatment.

### GSH and Reactive Oxygen Species Related Proteins

**Figure 10:**
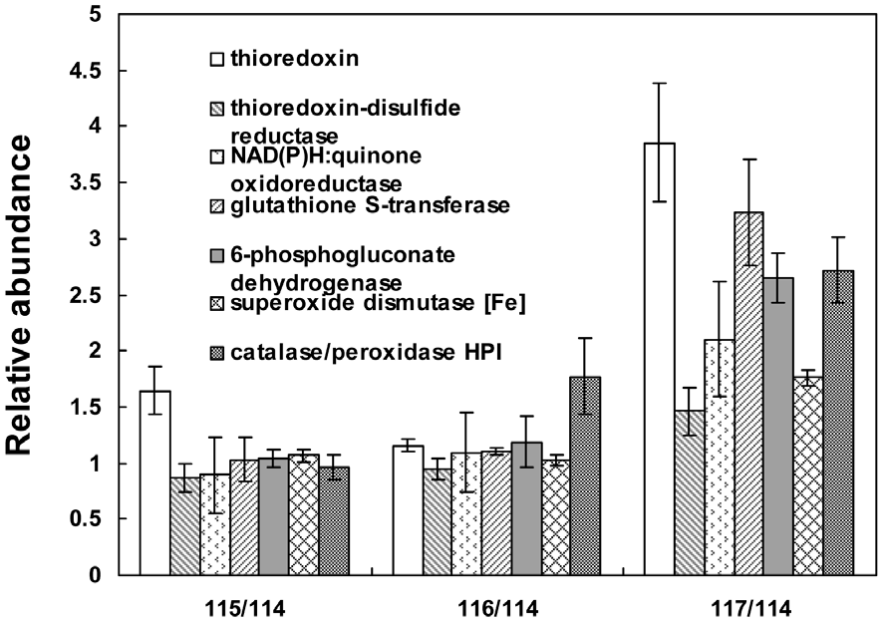
Profiles of proteins identified in combating against intracellular ROS.

GSH is the major nonprotein thiol compound in living cells, including human, yeast, and microorganism cells [35], [36], [37], which plays an important role in protecting cells against oxidative damage, toxic compounds, radiation and heavy metals [38]. Oxidation stress is caused by intracellular ROS, including superoxide anion, hydrogen peroxide, and hydroxyl radical as a result of oxygen metabolism in aerobic organisms [39]. ROS can cause damages to cells by attacking DNA, resulting in chain breaks, and modification of the carbohydrate parts and nitro bases [40]. In addition, ROS can also modify amino acids in proteins [39]. Several systems have been described to combat ROS in microorganisms, including catalase, superoxide dismutase, GSH metabolism related enzyme, thioredoxin and glutaredoxin [41]. In this study, we found that seven proteins involved in ROS detoxification were up-regulated after adding GSH in *K. vulgare* (Figure 10). Relative abundance variations of these seven proteins in the GSH treated *K. vulgare* compared with the control at 15 h of fermentation were as follows: superoxide dismutase, which reduces superoxide anion into molecular oxygen, had a 1.7-fold up-regulation; catalase, which reduces hydrogen peroxide into water and oxygen [41], had a 1.5-fold up-regulation; thioredoxin, thirodoxin-disulfide reductase and glutathione S-transferase respectively had a 3.3-fold, a 1.5-fold and a 2.9-fold up-regulation; 6-phosphogluconate dehydrogenase and NAD(P)H: quinine oxidoreductase had a 2.6-fold and a 1.9-fold up-regulation, respectively. It is well known that NADPH is the principal reducing power in cells, playing essential roles in GSH and thioredoxin metabolism [42]. The source of NADPH is glucose-6-phosphate dehydrogenase and 6-phosphogluconate dehydrogenase in the PPP [43]. Our results provided a speculation that the enhancement in PPP resulting from improved TPP transport, could generate more NADPH, which could then be used in the reduction of oxidized thioredoxin and GSSG, and in this way helped to combat against intracellular ROS.

As an aerobic bacterium, *K. vulgare* was inevitably subjected to ROS stress. In our former research, proteins for combating ROS stress in *K. vulgare* had similar up-regulation after the cell lysis of *B. megaterium* [18]. This coincidence highlighted the essential requirement for a reducing environment for the cells to grow and metabolize. In the case of this study, the positive effect of GSH also reflected in enhanced TPP transport and other processes associated with cell membranes of *K. vulgare*.

**Figure 11:**
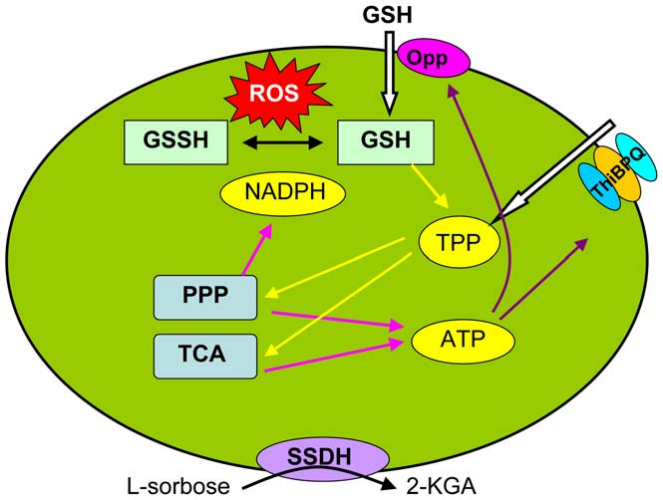
A proposed mechanism of GSH influencing *K. vulgare*: yellow arrow means positive effect; pink arrow means generation; white arrow means transport; black arrow means conversion; brown arrow means ATP supply. The transport of GSH into cell by the Opp system, improved the TPP transport, and enhanced the PPP and TCA cycle, thus generated more ATP and NADPH, which could then be used in solute transport and intracellular ROS depletion.

In this study, we aim to explore the mechanism of GSH's positive effect on *K. vulgare* by determine proteomic changes of *K. vulgare* when subjected to the reduced GSH. We generated comparative proteomic profiles for *K. vulgare* with and without GSH addition during the time course of the fermentation. Although no distinctive changes at early fermentation phase (1 h), at 15 h of the fermentation, the cells of the test group had a 4–6-fold increase in cell length, and the proteome profiles of *K. vulgare* showed significant differences. A proposed mechanism of GSH to *K. vulgare* was illustrated in Figure 11. In response to the GSH stimulation, the expressions of several proteins involved in cell membrane function, including oligopeptide transport protein and thiamin/TPP transport protein, membrane-bound dehydrogenase and outer membrane integrity maintainer were significantly increased. The coenzyme TPP obtained by thiamin transport would enhance the enzymatic activities of PPP and TCA cycle, and thus generate more reducing power in the form of NADPH and energy in the form of ATP, which could then be used in combating against intracellular ROS. This work provides valuable information regarding the molecular mechanism of GSH's enhancing effects on *K. vulgare* growth and 2-KGA production, and reveals the demand of *K. vulgare* for substrate transport for thiamin/TPP and oligopeptides, and the demand for antioxidant protection. Similar antioxidant protection requirement in *K. vulgare* also occurred in the mix-culture of *K. vulgare* and *B. megaterium*, and this coherence emphasized the importance of a reducing environment to *K. vulgare*, thus this requirement could be potential target in *K. vulgare* for future gene modification, which may eventually lead to the establishment of high-efficiency mono-strain fermentation for vitamin C production in industry.

## Materials and Methods

### Cell Culture and Growth Conditions

The strain of *K. vulgare* was kindly provided by Prof. Yuezhong Li of Shandong University in China. It was first inoculated in a seed medium containing: 20 g/L L-sorbose, 1 g/L KH_2_PO_4_, 3 g/L yeast powder, 0.2 g/L MgSO_4_, 3 g/L beef extract, 1 g/L urea, 10 g/L peptone, and 3 g/L corn steep liquor. The seed was then transferred to the fermentation medium with a 10% inoculum. The fermentation medium contains: 80 g/L L-sorbose, 20 g/L corn steep liquor, 1 g/L KH_2_PO_4_, 0.2 g/L MgSO_4_, and 12 g/L urea. The pH values of seed and fermentation media were adjusted to 6.8 and 7.0, respectively using 5 M NaOH before cultivation. *K. vulgare* was cultivated in a 250 mL flask with a 50 mL fermentation volume, shaken at a speed of 250 rpm at 30°C for about 70 h. GSH was added into the test group at the beginning of the fermentation with a final concentration of 1 g/L, and other cultivation conditions were identical as the control group.

### Analyses of 2-KGA and L-sorbose

The analyses of extracellular 2-KGA and L-sorbose were performed by the use of High Performance Liquid Chromatography (Waters Corp., Massachusetts, USA) coupled with a refractive index detector. 5 mM H_2_SO_4_ was used as the eluent on an Aminex HPX-87H column (BioRad, CA) at the temperature of 65°C with a flow rate of 0.6 mL/min.

### Determination of Extracellular GSH

The concentration of GSH in the fermenting liquor was determined by LC-MS/MS using a Venusil XBP C_18_ column coupled with a LCQ DecaXP Maxa mass spectrometer (Thermo Finnigan, Palo Alto, CA). A 30-min elution gradient for the reverse-phase chromatography separation consisting of a 3-min of 100% buffer A (5% acetonitrile, 0.1% formic acid in water); a 4-min gradient to 95% buffer B (0.1% formic acid in acetonitrile) followed by a 5-min maintenance; a 2-min gradient to 100% buffer A; and a 16-min re-equilibration at 100% buffer A was adopted. Selected reaction monitoring (SRM) mode was chosen to perform the mass detection. It works by isolating the target parent ion with a mass to charge ratio of 308 in the form of [GSH+H]^+^, initiating collision induced dissociation (CID), detecting and quantifying its unique daughter ion with a 179 *m/z*, derived from the breakage of amide bond between glutamyl and cysteinyl groups, to achieve the precise quantification of parent ion. This mass detection mode has the virtue of confirmative quantification of chemicals at lower concentrations. A tolerance of 0.5 *m/z* was allowed. GSH content in the fermentation liquor was determined according to a GSH standard calibration curve.

### Scanning Electron Microscopy and Transmission Electron Microscopy of *K. vulgare*

The sample preparation method for scanning electron microscopy was according to Ridgway [44] with minor modifications. 5 mL of bacteria suspensions was subjected to a 5000× g centrifugation at 4°C for 5 min and washed twice with phosphate buffer solution (PBS, pH 7.0) to obtain the cells for subsequent glutaraldehyde fixation and ethanol gradient dehydration. *K. vulgare* cells were fixed in 3% (w/v) phosphate-buffered glutaraldehyde for 2 h. Then a 5-min centrifugation was applied at a speed of 5000× g, followed by twice washes with distilled H_2_O. Increasing concentrations of ethanol in water (30%, 50%, 70%, 90%, 100%) were used in dehydration of the cells with each concentration lasting for 10 min. Dehydrated cells were dried on a SpeedVac, mounted on metal stubs with silver paint, and coated with gold. Finally, specimens were observed in a scanning electron microscope (Nova NanoSEM 430, FEI.).

For transmission electron microscopy observation, the cells were first fixed in 3% glutaraldehyde for 4 h, and then in 1% osmium tetroxide for postfixation. After dehydration in graded ethanol as described before, the cells were embedded in SPI-PON 812 resin, sectioned with a LKBV, mounted on uncoated grids, stained with uranyl acetate followed by lead citrate, and examined with Philips EM400ST microscope.

### Sampling, Extracting of Proteins and iTRAQ Labelling

*K. vulgare* cells with and without GSH were harvested at 1 h and 15 h of fermentation, the time points that respectively represented the beginning and middle phases of extracellular GSH depletion according to our preliminary experiment result, by a centrifugation at 5000× g for 5 min at 4°C. The supernatants were collected for 2-KGA, L-sorbose and GSH analyses. Meanwhile, the pellets were quenched by liquid nitrogen after washing twice with PBS (pH 7.2) and once with distilled water.

Extraction of proteins was conducted according to Cheng [45] with minor modifications. First, 0.1 g cells were grounded in liquid nitrogen with mortar and pestle, suspended in 0.5 mL of lysis buffer (8 M urea, 4% m/v CHAPS, 40 mM Tris-HCl, and 1 mM PMSF) on ice, and then vortexed. Next, the suspensions went through a pulsed ultrasonication for 8 times, each time lasting for 6 s followed by a 10 s pause, and then were stored at 4°C for 2 h. After centrifugation at 12000× g for 40 min at 4°C, the supernatants were transferred to new tubes, and protein contents were determined using the Bradford method [46]. Then a total of 100 µg proteins were precipitated using 4 times volume of ice-cold acetone at −40°C overnight. Finally, following twice washes of the precipitant with 80% cold acetone in water, the pellets were dried on a SpeedVac, waiting for iTRAQ labelling.

The protein pellets were dissolved in 20 µL of dissolution buffer (50 mM TEAB) with 1 µL of denaturant (0.1% SDS), reduced by 2 µL of reducing agent (50 mM TCEP) and incubated at 60°C for 1 h according to the iTRAQ labelling protocol supplied by the manufacturer (Applied Biosystems) and then cysteine blocked using 1 µL of MMTS at room temperature for 10 min. The following digestion was carried out by adding 20 µL of 0.25 µg/µL sequence grade modified trypsin (Promega, Madison WI, USA) solution to each sample at 37°C overnight for about 18 h. iTRAQ tags 114, 115, 116, 117 were labelled as follows: *K. vulgare* at 1 h (Kv-1 h) = iTRAQ 114; *K. vulgare* with GSH addition at 1 h (GSH-1 h) = iTRAQ 115; *K. vulgare* at 15 h (Kv-15 h) = iTRAQ 116; *K. vulgare* with GSH addition at 15 h (GSH-15 h) = iTRAQ 117. The labelled samples were then pooled, concentrated on a SpeedVac, diluted with strong cation exchange (SCX) buffer A (5% acetonitrile in water, 0.1% formic acid) to an approximate peptide concentration of 1 µg/µL for further 2-D nano LC-MS/MS analysis.

### On-line 2-D Nano-LC and Mass Spectrometry Analysis

An Agilent 1200 series nanoflow LC system (Agilent Technologies, USA) interfaced with an electrospray ionization micro-Q-TOF II mass spectrometer (Bruker Daltonics, Germany) was applied for the peptide separation and detection. First, 5 µL of peptide mixture was loaded onto a ZORBAX BIO-SCX II column (3.5 µm, 35×0.3 mm) by SCX buffer A at a flow of 10 µL/min pumped by a capillary pump, and was then eluted onto a ZORBAX 300SB-C_18_ trap column (5 µm, 5×0.3 mm) by sequentially injecting 5 µL increasing concentrations of ammonium chloride (NH_4_Cl) solution (2 mM, 10 mM, 20 mM, 40 mM, 60 mM, 80 mM, 100 mM, 300 mM, 500 mM, 1 M) to achieve the first dimension separation and the enrichment. In the second dimension separation, a ten-port valve for the switch of the first and second dimension separation switched the trap column into the solvent path of the nanopump, and the peptides retained on the trap column were then eluted by a gradient eluent consisted of buffer B (5% acetonitrile in water, 0.1% formic acid) and buffer C (5% water in acetonitrile, 0.1% formic acid) at a flow of 300 nL/min on a ZORBAX 300SB-C_18_ analytical column (3.5 µm, 150 mm×75 µm). The 120-min reverse-phase gradient elution contained: a 5-min of buffer A; a 10-min linear ramp to 10% buffer B; a 70-min ramp to 35% buffer B; a 5-min ramp to 80% buffer B; a 8-min ramp to 90% buffer B; a 3-min ramp back to 100% buffer A; and a 19-min re-equilibration of 100% buffer A.

After LC separation, mass data acquisitions were conducted using micrOTOFcontrol 3.0 (Bruker Daltonics, Germany). The electrospray of ions was generated by applying a 1500 V high voltage on the ionization tip (10±1 µm, SilicaTip), with a 150°C interface temperature and a 2 L/min flow of dry gas. A mass range between 1200 and 2500 *m/z* was acquired with 5 most abundant precursors selected for further MS/MS analysis, and the fragments covered a mass range between 70 and 2500 *m/z*. Each peptide was selected twice and then dynamically excluded for 0.2 min to have more peptides be detected.

### Database Searching and Analysis

The mass spectrums after smoothed and background subtracted were analyzed by the tool of DataAnalysis (Bruker Daltonics, Germany). After finding auto MS (n) peak lists and deconvouluting the mass spectra, compound lists were exported in mgf files. Different mgf files from different runs of NH_4_Cl solution elution were combined into one file and was then used in protein identification using the Mascot database search algorithm with the threshold set at *p*<0.05. The database was an in-house protein database for *K. vulgare* with 3179 sequences. The searching parameters were set as follows: one maximum missed cleavage of trypsin; fixed modifications including MMTS modification of cysteine (Methylthio (C)), iTRAQ 4-plex (N-term) and iTRAQ 4-plex (K); variable modifications consisting of oxidation of methionine and iTRAQ 4-plex (Y); 0.1 Da peptide mass tolerance; 0.05 Da fragment mass tolerance. We required a match of at least two peptides per protein as a confident detection. The quantification of labelled proteins was conducted also by the Mascot server by performing the iTRAQ 4-plex quantification. Three biological replicates were labelled and analyzed in parallel. InterPro (http://www.ebi.ac.uk/interpro/) was used for protein sequence analyses and classification, and the Kyoto Encyclopedia of Genes and Genomes (http://www.genome.jp/kegg/pathway.html) was used to reconstruct major metabolic pathways of *K. vulgare*. Database of Clusters of Orthologous Groups of proteins (http://www.ncbi.nlm.nih.gov/COG) [47], [48] was utilized to perform phylogenetic classification of proteins. After mean centering, the data of protein abundances was subjected to PCA analysis by Matlab 7.0.

### Extraction, 2-DE, MALDI-TOF Identification of Glutathione Reductase

Cells of *K. vulgare* with and without GSH at 15 h were harvested, respectively. Extraction of proteins and 2-DE were performed according to the method described before [18]. Each experiment was carried out at least twice for replicates. After calculating the theoretical molecular weight and PI of glutathione reductase by ExPASy Compute pI/Mw tool (http://web.expasy.org/compute_pi/), we found the possible location area of GR on gel according to the calculated Mw of and PI of 5.24. Then peptide mass fingerprint (PMF) identification was performed by Autoflex TOF-TOF II (Bruker Daltonics, Germany) for several spots around this area. Mascot PMF searching was performed with a required peptide mass tolerance of ±100 ppm. Finally, we identified glutathione reductase with 6 matches of peptides, 22% protein coverage and a mascot score of 65, and ultimately we found its exact location on gel.

## Supporting Information

Table S1 Proteins identified and quantified by iTRAQ-2-D-LC-MS/MS.
(DOC)

Figure S1 Growth and 2-KGA production of *K. vulgare* after adding thiamin: (A) Optical density of *K. vulgare* after adding thiamin compared with control grown with and without GSH; (B) 2-KGA production of *K. vulgare* at 72 h after adding thiamin compared with control grown with and without GSH.
(TIF)

## References

[1] Szent-Gyorgyi A (1928). Observations on the functions of peroxidase systems and the chemistry of the adrenal cortex.. Biochem Jour.

[2] Sauberlich HE (1994). Pharmacology of vitamin C.. Annu Rev Nutr.

[3] Boudrant J (1990). Microbiol processes for ascorbic acid biosynthesis: a review.. Enzyme Microb Technol.

[4] Yin GL, Tao ZX, Yan ZZ, Ning WZ, Wang CH (1990). Fermentation process.. United States Patent.

[5] Li ZJ, Ji XJ, Kan SL, Qiao HQ, Jiang M (2010). Past, present, and future industrial biotechnology in China.. Adv Biochem Engin/Biotechnol.

[6] Bremus C, Herrmann U, Meyer SB, Sahm H (2006). The use of microorganisms in L-ascorbic acid production.. J Biotechnol.

[7] Takagi Y, Sugisawa T, Hoshino T (2010). Continuous 2-keto-L-gulonic acid fermentation by mixed culture of *Ketogulonicigenium vulgare* DSM 4025 and *Bacillus megaterium* or *Xanthomonas maltophilia*.. Appl Microbiol Biotechnol.

[8] Zhang J, Zhou JW, Liu J, Chen KJ, Liu LM (2011). Development of chemically defined media supporting high cell density growth of *Ketogulonicigenium vulgare* and *Bacillus megaterium*.. Bioresour Technol.

[9] Urbance JW, Bratina BJ, Stoddard SF, Schmidt TM (2001). Taxonomic characterization of *Ketogulonigenium vulgare* gen. nov., sp. nov. and *Ketogulonigenium robustum* sp. nov., which oxidize L-sorbose to 2-keto-L-gulonic acid.. Int J Syst Evol Microbiol.

[10] Eaton MW, Ellar DJ (1974). Protein synthesis and breakdown in the mother-cell and forespore compartments during spore morphogenesis in *Bacillus megaterium*.. Biochem J.

[11] Leduc S, de Troostembergh JC, Lebeault JM (2004). Folate requirements of the 2-keto-L-gulonic acid-producing strain *Ketogulonigenium vulgare* LMP P-20356 in L-sorbose/CSL medium.. Appl Microbiol Biotechnol.

[12] Yuan YJ, Yi H, W LL, Zhou J, Ma Q (2009). A method of increased production of 2-keto-L-gulonic acid by *Gluconobacter oxydans*.. China Patent.

[13] Miyazaki T, Sugisawa T, Hoshino T (2006). Pyrroloquinoline quinone-dependent dehydrogenases from *Ketogulonicigenium vulgare* catalyze the direct conversion of L-sorbosone to L-ascorbic acid.. Appl Environ Microbiol.

[14] Claus GW, Batzing BL, Baker CA, Goebel EM (1975). Intracytoplasmic membrane formation and increased oxidation of glycerol during growth of *Gluconobacter oxydans*.. J Bacteriaol.

[15] White SA, Claus GW (1982). Effect of intracytoplasmic membrane development on oxidation of sorbitol and other polyols by *Gluconobacter oxydans*.. J Bacteriaol.

[16] Lin FM, Qiao B, Yuan YJ (2009). Comparative proteomic analysis of tolerance and adaptation of ethanologenic *Saccharomyces cerevisiae* to furfural, a lignocellulosic inhibitory compound.. Appl Environ Microbiol.

[17] Lin FM, Tan Y, Yuan YJ (2009). Temporal quantitative proteomics of *Saccharomyces cerevisiae* in response to a nonlethal concentration of furfural.. Proteomics.

[18] Ma Q, Zhou J, Zhang WW, Meng XX, Sun JW (2011). Integrated proteomic and metabolomic analysis of an artificial microbial community for two-step production of vitamin C.. PLoS ONE.

[19] Heefner DL, Claus GW (1976). Change in quantity of lipids and cell size during intracytoplasmic membrane formation in *Gluconobacter oxydans*.. J Bacteriaol.

[20] Zhou J, Ma Q, Yi H, Wang LL, Song H (2011). Metabolome profiling reveals metabolic cooperation between *Bacillus megaterium* and *Ketogulonigenium vulgare* during induced swarm motility.. Appl Environ Microbiol.

[21] Webb E, Claas K, Downs D (1998). *thi*BPQ encodes an ABC transporter required for transport of thiamine and thiamine pyrophosphate in *Salmonella typhimurium*.. J Biol Chem.

[22] Sudarsan N, Chalamish SC, Nakamura S, Emilsson GM, Breaker RR (2005). Thiamine pyrophosphate riboswitches rre targets for the antimicrobial compound pyrithiamine.. Chem Biol.

[23] Zangen A, Shainberg A (1997). Thiamine deficiency in cardiac cells in culture.. Biochem Pharmacol.

[24] McCandless DW (1982). Energy metabolism in the lateral vestibular nucleus in pyrithiamin-induced thiamin deficiency.. Ann N Y Acad Sci.

[25] Hancock RD (2009). Recent patents on vitamin C: opportunities for crop improvement and single-step biological manufacture.. Recent Pat Food Nutr Agric.

[26] Rindi G, Ferrari G, Ventura U, Trotta A (1966). Action of amprolium on the thiamine content of rat tissues.. J Nutr.

[27] Winkler W, Nahvi A, Breaker RR (2002). Thiamine derivatives bindmessenger RNAs directly to regulate bacterial gene expression.. Nature.

[28] Solomon J, Su L, Shyn S, Grossman AD (2003). Isolation and characterization of mutants of the *Bacilllus subtilis* oligopeptide permease with altered specificity of oligopeptide transport.. J Bacteriaol.

[29] Tynkkynen S, Buist G, Kunji E, Kok J, Poolman B (1993). Genetic and biochemical characterization of the oligopeptide transport system of *Lactococcus lactis*.. J Bacteriaol.

[30] Lazzaroni JC, Dubuisson JF, Vianney A (2002). The Tol protein of *Escherichia coli* and their involvement in the translocation of group A colicins.. Biochimie.

[31] Germon P, Ray MC, Vianney A, Lazzaroni JC (2001). Energy-dependent conformational change in the TolA protein of *Escherichia coli* involves its N-terminal domain, TolQ, and TolR.. J Bacteriaol.

[32] Muller MM, Vianney A, Lazzaroni JC, Webster RE, Portalier R (1993). Membrane topology of the *Escherichia coli* TolR protein required for cell envelope integrity.. J Bacteriaol.

[33] Zhang WC, Jiao YH, Yuan HJ, Xie L (2003). A novel L-sorbose dehydrogenase gene and the encoding protein.. China Patent.

[34] Fu SL, Zhang WC, Guo AG, Wang JH (2007). Identification of promoters of two dehydrogenase genes in *Ketogulonicigenium vulgare* DSM 4025 and their strength comparison in *K. vulgare* and *Escherichia coli*.. Appl Microbiol Biotechnol.

[35] Li Y, Hugenholtz J, Abee T, Molenaar D (2003). Glutathione protects *Lactococcus lactis* against oxidative stress.. Appl Environ Microbiol.

[36] Dickinson DA, Forman HJ (2002). Cellular glutathione and thiols metabolism.. Biochem Pharmacol.

[37] Smirnova GV, Muzyka NG, Glukhovchenko MN, Oktyabrsky ON (2000). Effects of menadione and hydrogen peroxide on glutathione status in growing Escherichia coli.. Free Radic Biol Med.

[38] Meister A, Anderson ME (1983). Glutathione.. Ann Rev Biochem.

[39] Lushchak VI (2001). Oxidative stress and mechanisms of protection against it in bacteria.. Biochemistry.

[40] Halliwell B, Gutteridge JMC (1989).

[41] Temple MD, Perrone GG, Dawes IW (2005). Complex cellular responses to reactive oxygen species.. Trends Cell Biol.

[42] Prinz WA, Aslund F, Holmgren A, Beckwith J (1997). The role of the thioredoxin and glutaredoxin pathways in reducing protein disulfide bonds in the *Escherichia coli* cytoplasm.. J Biol Chem.

[43] Bozdech Z, Ginsburg H (2005). Data mining of the transcriptome of *Plasmodium falciparum*: the pentose phosphate pathway and ancillary processes.. Malar J.

[44] Ridgway HF, Olson BH (1981). Scanning electron microscope evidence for bacterial colonization of a drinking-water distribution system.. Appl Environ Microbiol.

[45] Cheng JS, Zhou X, Ding MZ, Yuan YJ (2009). Proteomic insights into adaptive responses of *Saccharomyces cerevisiae* to the repeated vacuum fermentation.. Appl Microbiol Biotechnol.

[46] Bradford MM (1976). A rapid and sensitive method for the quantitation of microgram quantities of protein utilizing the principle of protein-dye binding.. Anal Biochem.

[47] Tatusov RL, Galperin MY, Natale DA, Koonin EV (2000). The COG database: a tool for genome-scale analysis of protein functions and evolution.. Nucleic Acids Res.

[48] Tatusov RL, Natale DA, Garkavtsev IV, Tatusova TA, Fedorova ND (2001). The COG database: new developments in phylogenetic classification of proteins from complete genomes.. Nucleic Acids Res.

