# Supplementary material for: Proteomic Analysis of Ketogulonicigenium vulgare under Glutathione Reveals High Demand for Thiamin Transport and Antioxidant Protection
Source: PLoS One. 2012 Feb 22;7(2):e32156. doi: 10.1371/journal.pone.0032156 (PMC3284542; doi:10.1371/journal.pone.0032156)
Supplement: Table S1 — Proteins identified and quantified by iTRAQ-2-D-LC-MS/MS. (DOC) [file pone.0032156.s001.doc]

**Table S1 Proteins identified and quantified by iTRAQ-2-D-LC-MS/MS**

| **Protein name** | **Protein score** | **Matches of sequences** | **Protein coverage (%)** | **Protein PI** | **115/114** | **116/114** | **117/114** |
| --- | --- | --- | --- | --- | --- | --- | --- |
| glutamate synthase [NADPH] large chain | 22 | 4 | 2.1 | 5.97 | 1.058 | 1.105 | 1.848 |
| glutamyl-tRNA(Gln) and/or aspartyl-tRNA(Asn) amidotransferase, A subunit | 40 | 3 | 6.7 | 5.06 | 1.018667 | 1.136 | 2.523333 |
| D-alanyl-D-alanine carboxypeptidase family protein | 33 | 2 | 2 | 5.04 | 1.098 | 0.738 | 1.596 |
| bacterial extracellular solute-binding proteins, family 5 Middle family protein | 651 | 4 | 8.8 | 4.25 | 1.069 | 1.120333 | 2.787667 |
| uncharacterized ABC transporter ATP-binding protein yejF | 32 | 4 | 7.5 | 7.21 | 1.273 | 1.314667 | 2.961333 |
| sulfate adenylyltransferase subunit 2 (Sulfate adenylatetransferase) (SAT) (ATP-sulfurylase small subunit) | 20 | 2 | 6.3 | 6.61 | 0.997 | 1.354 | 2.43 |
| putative N-acetylmannosamine-6-P epimerase | 42 | 2 | 5.4 | 4.94 | 1.179 | 1.299 | 2.3505 |
| coenzyme PQQ biosynthesis protein C | 70 | 4 | 14.6 | 6.63 | 1.050667 | 1.057 | 1.438333 |
| glutamine transport ATP-binding protein glnQ | 29 | 4 | 14 | 6.6 | 0.904667 | 0.748 | 0.894 |
| ribosomal protein L17 | 76 | 3 | 16.5 | 9.94 | 1.158 | 1.499333 | 4.547333 |
| DNA-directed RNA polymerase, alpha subunit | 65 | 7 | 16.9 | 4.69 | 1.035333 | 1.148333 | 2.451667 |
| 30S ribosomal protein S11 (BS11) | 53 | 2 | 21.5 | 10.98 | 1.2215 | 1.475 | 2.9275 |
| 30S ribosomal protein S13 | 85 | 3 | 23 | 10.89 | 1.037 | 1.085667 | 3.281333 |
| ribosomal protein L15 | 25 | 4 | 21.4 | 10.38 | 1.001 | 1.08 | 4.389 |
| ribosomal protein L30 | 50 | 3 | 48.4 | 10.87 | 1.161 | 1.527 | 4.390667 |
| ribosomal protein S5 | 39 | 5 | 22.5 | 10.99 | 1.098 | 1.084 | 2.221 |
| ribosomal protein L18 | 170 | 5 | 30.3 | 10.5 | 1.049 | 1.109333 | 2.605 |
| 50S ribosomal protein L6 (BL10) | 70 | 3 | 16.6 | 9.75 | 0.997 | 1.172333 | 2.766 |
| ribosomal protein S8 family protein | 38 | 4 | 23.1 | 9.6 | 1.148667 | 1.265667 | 3.210667 |
| 2-deoxy-D-gluconate 3-dehydrogenase | 30 | 2 | 4.3 | 6.11 | 0.865 | 0.811 | 1.790333 |
| ribosomal protein S14p/S29e family protein | 44 | 5 | 39.6 | 10.4 | 1.118333 | 1.063667 | 2.761667 |
| 50S ribosomal protein L5 (BL6) | 413 | 7 | 33.2 | 9.3 | 1.039333 | 1.127667 | 2.343667 |
| ribosomal protein L24 | 31 | 2 | 17.8 | 10.19 | 1.117 | 0.980333 | 2.240333 |
| ribosomal protein S17 family protein | 38 | 2 | 13.2 | 10.29 | 1.164 | 1.348 | 2.476 |
| ribosomal protein L29 | 30 | 5 | 67.6 | 10.14 | 0.976333 | 1.267667 | 3.245 |
| ribosomal protein S3 | 242 | 8 | 24.4 | 10.02 | 1.085 | 1.161333 | 2.361333 |
| ribosomal protein L22 | 24 | 3 | 22.6 | 10.48 | 1.035333 | 1.067 | 2.528333 |
| ribosomal protein S19 | 194 | 4 | 40.2 | 10.17 | 1.113333 | 1.181333 | 2.170667 |
| ribosomal protein L2 | 206 | 6 | 26.6 | 10.79 | 1.028 | 0.935 | 1.97 |
| ribosomal protein L23 family protein | 174 | 3 | 28.6 | 9.82 | 1.035 | 1.029667 | 2.145333 |
| 50S ribosomal protein L4 (BL4) | 46 | 5 | 27.2 | 10.1 | 1.068667 | 1.301 | 3.578667 |
| ribosomal protein L3 family protein | 234 | 9 | 28.7 | 10.28 | 1.075333 | 1.164333 | 3.189667 |
| ribosomal protein S10 | 25 | 3 | 26.9 | 9.81 | 1.094333 | 1.567667 | 3.715 |
| translation elongation factor Tu | 6360 | 12 | 34 | 5.28 | 1.043333 | 1.140667 | 2.601 |
| translation elongation factor G | 102 | 14 | 16.9 | 4.98 | 0.995667 | 1.013333 | 2.324667 |
| ribosomal protein S7 | 195 | 4 | 27.6 | 9.93 | 1.191333 | 1.206667 | 2.942333 |
| DNA-directed RNA polymerase, beta subunit | 47 | 16 | 11 | 5.94 | 1.064667 | 1.119 | 3.127667 |
| DNA-directed RNA polymerase, beta subunit | 172 | 17 | 11.2 | 5.03 | 0.978667 | 1.142 | 2.527333 |
| ribosomal protein L7/L12 | 511 | 4 | 23.6 | 4.86 | 1.047 | 1.170667 | 2.335333 |
| ribosomal protein L1 | 128 | 3 | 16.8 | 9.36 | 1.258667 | 1.287 | 2.988 |
| ribosomal protein L11 | 31 | 2 | 10 | 9.85 | 1.049667 | 0.85 | 2.536 |
| transcription termination/antitermination factor NusG | 20 | 2 | 7.9 | 5.57 | 1.136333 | 1.269667 | 1.939333 |
| luciferase-like monooxygenase family protein | 13 | 3 | 7.6 | 6.73 | 1.32 | 0.957 | 3.177 |
| ribonuclease R | 21 | 5 | 4.9 | 6.77 | 0.8725 | 0.775 | 0.8895 |
| 2,3,4,5-tetrahydropyridine-2,6-dicarboxylate N-succinyltransferase | 171 | 2 | 5.8 | 5.27 | 1.114333 | 1.114 | 2.010333 |
| hypothetical protein | 19 | 2 | 1.9 | 8.85 | 0.813 | 1.662 | 2.233 |
| cytochrome c family protein | 84 | 2 | 5.4 | 4.65 | 1.174333 | 1.115667 | 2.341 |
| sorbose/sorbosone dehydrogenase | 1326 | 10 | 16.2 | 4.41 | 1.170333 | 1.099667 | 1.655667 |
| ABC transporter, periplasmic substrate-binding protein | 651 | 4 | 16.3 | 4.29 | 1.055333 | 0.967 | 1.908 |
| aminopeptidase 2 (Aminopeptidase II) (AP-II) | 29 | 2 | 5.1 | 5.31 | 1.066 | 0.952 | 1.862 |
| sorbose/sorbosone dehydrogenase | 830 | 10 | 17.6 | 4.38 | 1.173333 | 1.097667 | 1.566 |
| YCII-related domain protein | 24 | 2 | 12.4 | 5.54 | 1.252 | 0.962 | 2.36 |
| ribosomal subunit interface protein | 29 | 2 | 3.6 | 5.61 | 1.464 | 1.208 | 2.799 |
| arginine biosynthesis bifunctional protein ArgJ | 23 | 2 | 2 | 4.69 | 1.084 | 1.224 | 2.5945 |
| peptidase family M3 family protein | 20 | 2 | 1.6 | 4.97 | 1.681667 | 0.982 | 0.947667 |
| cobalamin-independent synthase, Catalytic domain protein | 347 | 10 | 26.1 | 5.59 | 1.075 | 1.090333 | 1.666667 |
| translation initiation factor IF-2 | 30 | 3 | 4.2 | 5.84 | 1.418 | 1.413333 | 3.709667 |
| zinc-binding dehydrogenase family protein | 27 | 2 | 5.5 | 5.62 | 1.0215 | 1.561 | 2.1715 |
| argininosuccinate synthase | 86 | 7 | 17.7 | 5.38 | 1.125 | 1.193667 | 2.209333 |
| transcription termination factor Rho | 27 | 5 | 10 | 5.08 | 0 | 2.245 | 2.274 |
| protein-export chaperone SecB | 29 | 3 | 13.1 | 5.26 | 1.179333 | 1.333 | 3.468333 |
| tim44-like domain protein | 29 | 2 | 4.1 | 4.83 | 1.261667 | 1.446667 | 2.113333 |
| thioredoxin | 83 | 2 | 23.1 | 4.62 | 1.648 | 1.156 | 3.850667 |
| double-strand break repair protein AddB | 22 | 3 | 3.2 | 5.93 | 0 | 4.834 | 0 |
| adenosylhomocysteinase | 26 | 9 | 14 | 5.54 | 0.959 | 1.1015 | 2.2285 |
| chain D, Crystal Structure Of Uncharacterized Protein | 180 | 7 | 46.9 | 8.73 | 1.347667 | 1.355333 | 2.878 |
| branched-chain amino acid aminotransferase | 25 | 3 | 9 | 5.75 | 1.013 | 1.0805 | 1.5535 |
| hypothetical protein | 113 | 3 | 9 | 3.91 | 1.054333 | 1.019667 | 1.783 |
| response regulator | 97 | 2 | 7.9 | 5.37 | 1.226667 | 1.119667 | 1.927 |
| uncharacterized protein-like protein | 19 | 2 | 11.2 | 11.1 | 1.276 | 1.625 | 2.917 |
| thiamin/thiamin pyrophosphate ABC transporter, thiamin/thiamin pyrophospate-binding protein | 139 | 3 | 13.1 | 4.02 | 1.265 | 1.441333 | 10.64767 |
| chorismate synthase | 16 | 2 | 6.5 | 6.08 | 0.873333 | 1.034667 | 2.354667 |
| DNA-binding protein HU 1 (DNA-binding protein II) (HB) | 61 | 3 | 33.7 | 9.61 | 0.998667 | 1.123 | 3.045333 |
| chaperone protein DnaJ | 65 | 2 | 5.2 | 7.57 | 1.016667 | 1.141333 | 2.053333 |
| chaperone protein DnaK | 1476 | 17 | 24.1 | 4.79 | 1.091 | 1.594667 | 2.073 |
| ribosomal protein S15 | 117 | 4 | 28.1 | 10.05 | 0.976667 | 1.036 | 2.360667 |
| S1 RNA binding domain protein | 99 | 9 | 10.4 | 5.17 | 1.150667 | 1.160333 | 2.717333 |
| hypothetical protein | 17 | 2 | 3.5 | 4.81 | 2.927 | 1.853 | 4.856 |
| D-isomer specific 2-hydroxyacid dehydrogenase, NAD binding domain protein | 21 | 2 | 8.7 | 5.15 | 0.8075 | 0.8365 | 1.831 |
| NAD(P)H:quinone oxidoreductase | 19 | 5 | 20.9 | 5.15 | 0.894 | 1.093 | 2.1015 |
| phosphoserine aminotransferase | 49 | 4 | 12.3 | 5.07 | 1.181667 | 0.952667 | 1.766667 |
| phosphoglycerate dehydrogenase | 319 | 13 | 27.1 | 5.33 | 1.115333 | 1.155667 | 1.953333 |
| ATP-dependent chaperone ClpB | 89 | 17 | 18.9 | 5.22 | 1.130333 | 1.792 | 2.125 |
| resolvase, N terminal domain protein | 23 | 3 | 4.1 | 9.43 | 1.2515 | 1.505 | 4.01 |
| antirestriction protein | 30 | 2 | 6.5 | 6.37 | 0.9445 | 0.9205 | 2.094 |
| uncharacterized ABC transporter ATP-binding protein yehX | 13 | 2 | 4.5 | 5.98 | 1.966 | 2.725 | 3.323 |
| AGR_L_2804p, nitrilotriacetate monooxygenase component A homolog ytnJ - Bacillus subtilis | 49 | 2 | 6.2 | 6.02 | 0.9005 | 0.6615 | 1.5065 |
| YKOF-related family protein | 59 | 2 | 6.4 | 5.28 | 1.49 | 1.431 | 7.557333 |
| glutathione S-transferase, C-terminal domain protein | 19 | 2 | 4.3 | 5.8 | 1.0315 | 1.1015 | 3.2325 |
| binding-protein-dependent transport system inner membrane component family protein | 13 | 2 | 3.4 | 10.3 | 0.621 | 0 | 1.424 |
| glutathione synthase | 27 | 2 | 6.4 | 5.6 | 1.238 | 1.495 | 2.451333 |
| acetyl-CoA carboxylase, carboxyl transferase, beta subunit | 24 | 3 | 7.4 | 7.05 | 0.713 | 0.673 | 1.759 |
| ompA family protein | 21 | 2 | 2.7 | 4.86 | 1.481 | 3.032 | 2.365 |
| nitrogen regulatory protein P-II 1 | 109 | 5 | 48.2 | 5.45 | 1.049 | 1.031 | 1.875667 |
| dihydrodipicolinate synthase | 38 | 2 | 3.8 | 5.61 | 1.503 | 1.434 | 2.036 |
| aldo/keto reductase family protein | 142 | 6 | 18.8 | 5.01 | 1.083333 | 1.084333 | 2.370333 |
| DNA-directed RNA polymerase, omega subunit | 45 | 2 | 20.5 | 4.39 | 0.553 | 0 | 1.726 |
| aldo/keto reductase family protein | 21 | 6 | 11 | 6.06 | 1.0305 | 1.083 | 2.0495 |
| riboflavin biosynthesis protein RibD | 19 | 2 | 3.4 | 9.29 | 0 | 5.375 | 0 |
| 6,7-dimethyl-8-ribityllumazine synthase | 30 | 2 | 11.8 | 5.52 | 0.69 | 0.808 | 2.076 |
| aldo/keto reductase family protein | 28 | 3 | 9.8 | 6.25 | 1.1885 | 1.673 | 4.4825 |
| periplasmic binding proteins and sugar binding domain of the LacI family protein | 22 | 2 | 4.5 | 6.5 | 1.359667 | 0.974667 | 0.506667 |
| putative non-heme chloroperoxidase (Chlorideperoxidase) | 28 | 2 | 5.2 | 5 | 1.023 | 1.033 | 1.9135 |
| ATPase | 35 | 2 | 2.2 | 6.37 | 1.045667 | 1.267 | 2.442 |
| carbamoyl-phosphate synthase large chain (Carbamoyl-phosphate synthetase ammonia chain) | 35 | 2 | 1.6 | 4.82 | 1.169 | 1.0775 | 2.6785 |
| ribosomal protein S1 | 392 | 17 | 26.9 | 5.02 | 1.058333 | 1.168 | 2.592667 |
| integration host factor, beta subunit | 57 | 3 | 21.5 | 9.15 | 1.036333 | 1.383333 | 4.806667 |
| ribosomal protein L25, Ctc-form | 276 | 2 | 14.8 | 5.66 | 1.231 | 1.271667 | 2.777667 |
| L-lactate dehydrogenase [cytochrome] | 16 | 2 | 3.6 | 9.41 | 1.693 | 1.618 | 3.244 |
| membrane-bound aldehyde dehydrogenase [pyrroloquinoline-quinone] (ALDH) | 199 | 7 | 7.9 | 4.7 | 1.108333 | 1.064667 | 2.293667 |
| glutamine synthetase, catalytic domain protein | 18 | 2 | 2.7 | 5.02 | 1.28 | 1.619 | 2.707 |
| isoquinoline 1-oxidoreductase subunit alpha | 69 | 2 | 8.9 | 4.76 | 0.809 | 4.92 | 2.01 |
| adenylosuccinate synthetase | 18 | 3 | 6.5 | 5.34 | 1.183333 | 1.037667 | 2.136 |
| modulator of DNA gyrase family protein | 22 | 2 | 4.9 | 5.26 | 1.223 | 1.96 | 4.568 |
| cytochrome c oxidase, subunit II | 25 | 2 | 3.5 | 4.44 | 1.65 | 0.962 | 2.18 |
| hydantoinase/oxoprolinase family protein | 65 | 2 | 1.7 | 4.93 | 1.015 | 0.985 | 1.723 |
| threonyl-tRNA synthetase | 27 | 2 | 2.5 | 5.6 | 1.386 | 1.101 | 2.37 |
| mgpS | 48 | 2 | 2.8 | 8.91 | 1.233 | 1.124 | 2.3195 |
| hflC protein | 37 | 4 | 12.4 | 5.04 | 0.717 | 0.844 | 1.465 |
| protease Do family protein | 257 | 2 | 2.6 | 4.31 | 1.13 | 0.884667 | 0.986667 |
| immunoreactive 28 kDa outer membrane protein | 34 | 3 | 14.4 | 4.29 | 0.927 | 1.120667 | 2.308 |
| 6-phosphogluconate dehydrogenase, decarboxylating | 133 | 3 | 8.2 | 5.07 | 1.041333 | 1.188333 | 2.653 |
| ribosomal protein L31 | 18 | 2 | 26 | 6.81 | 1.192 | 1.116 | 2.303333 |
| prolyl-tRNA synthetase | 18 | 5 | 9.2 | 5.51 | 0.431 | 1.733 | 3 |
| ribosomal protein L19 | 43 | 4 | 33.6 | 10.35 | 1.180667 | 1.311667 | 2.581 |
| hypothetical protein | 207 | 3 | 31.9 | 4.43 | 1.139667 | 1.308333 | 2.038333 |
| glutamyl-tRNA synthetase | 25 | 3 | 6.3 | 5.28 | 0.989 | 1.091 | 2.165 |
| Cold-shock DNA-binding domain protein | 401 | 7 | 77.2 |  | 1.071333 | 1.079667 | 2.532667 |
| lysyl-tRNA synthetase | 44 | 2 | 2.1 | 5.28 | 1.128 | 1.019 | 2.637 |
| urease, alpha subunit | 18 | 2 | 1.2 | 5.9 | 0.917 | 0.973 | 1.502 |
| ribosomal protein S16 | 124 | 3 | 28.1 | 10.1 | 1.072333 | 1.161667 | 2.590333 |
| urease accessory protein UreG | 85 | 2 | 4.4 | 4.84 | 0.971667 | 0.923 | 1.975 |
| putative lipoprotein | 21 | 2 | 18 | 6.34 | 1.1385 | 1.383 | 2.259 |
| uroporphyrinogen decarboxylase | 26 | 2 | 3.5 | 5.22 | 1.346 | 1.319 | 2.038 |
| ribosomal protein S4 | 52 | 6 | 31.3 | 9.69 | 1.074333 | 1.189333 | 3.135 |
| hydroxyacylglutathione hydrolase | 52 | 3 | 12.1 | 5.98 | 1.017667 | 1.162667 | 2.074 |
| ATP synthase F1, delta subunit | 29 | 2 | 12.8 | 8.96 | 1.139667 | 1.196333 | 2.238333 |
| ATP synthase F1, alpha subunit | 933 | 22 | 40.2 | 5.89 | 1.059333 | 1.149333 | 2.541667 |
| ATP synthase F1, beta subunit | 1199 | 10 | 25.2 | 4.91 | 0.981333 | 1.084333 | 2.493333 |
| ATP synthase F1, epsilon subunit | 31 | 4 | 31.2 | 4.4 | 1.260667 | 1.302333 | 3.346 |
| 10 kDa chaperonin 1 (Protein Cpn10 1) (groES protein 1) | 1866 | 7 | 63.2 | 5.2 | 1.077667 | 1.214333 | 1.749667 |
| chaperonin GroL | 4256 | 23 | 40.4 | 4.97 | 1.113 | 1.321667 | 2.172 |
| thioredoxin-disulfide reductase | 28 | 2 | 3.5 | 5.21 | 0.866 | 0.942 | 1.464 |
| bacterioferritin | 358 | 4 | 26.7 | 4.82 | 1.011667 | 0.968 | 1.331 |
| ATP synthase B/B' CF(0) family protein | 31 | 2 | 12.4 | 4.61 | 1.022 | 1.063 | 2.004 |
| ketol-acid reductoisomerase | 33 | 3 | 10 | 5.57 | 0.787 | 0.72 | 2.096 |
| yqey-like family protein | 16 | 2 | 11.8 | 5.33 | 1.415 | 1.446 | 3.86 |
| O-succinylhomoserine sulfhydrylase | 16 | 2 | 2.6 | 5.33 | 1.368 | 0.906 | 2.44 |
| bacterial extracellular solute-binding proteins, family 5 Middle family protein | 28 | 2 | 4 | 4.24 | 0.8515 | 1.2105 | 2.943 |
| basic membrane family protein | 53 | 2 | 3.5 | 4.38 | 1.184333 | 1.233333 | 2.944667 |
| delta-aminolevulinic acid dehydratase (Porphobilinogensynthase) (ALAD) (ALADH) | 46 | 2 | 6 | 4.91 | 1.4845 | 1.258 | 2.2305 |
| beta-ketoacyl synthase, C-terminal domain protein | 50 | 3 | 7.8 | 5.6 | 1.054 | 1.140667 | 1.952 |
| invasion associated locus B (IalB) family protein | 29 | 2 | 12.2 | 4.27 | 1.311 | 1.53 | 2.8755 |
| single-stranded DNA-binding protein (SSB) (Helix-destabilizingprotein) | 32 | 2 | 5.1 | 5.92 | 1.132 | 1.492 | 3.117 |
| his Kinase A (phosphoacceptor) domain protein | 32 | 2 | 2.4 | 5.64 | 1.266 | 0.852 | 0.523 |
| enoyl-[acyl-carrier-protein] reductase [NADH] 1 (NADH-dependent enoyl-ACP reductase 1) | 181 | 7 | 23.5 | 5.23 | 1.065 | 1.131 | 1.938333 |
| cold shock protein cspB (Major cold shock protein) | 18 | 2 | 7.4 | 5.93 | 1.168 | 1.236 | 1.481 |
| sorbose/sorbosone dehydrogenase | 112 | 5 | 8.1 | 4.25 | 1.055 | 0.968667 | 3.638667 |
| trigger factor | 445 | 8 | 17.8 | 4.68 | 1.106667 | 1.053667 | 2.963333 |
| ribosomal protein L9 | 48 | 5 | 28.8 | 4.73 | 1.068667 | 1.079333 | 2.160333 |
| ribosomal protein S18 | 35 | 3 | 37.3 | 11.02 | 0.699 | 0.377 | 1.969 |
| ribosomal protein S6 | 130 | 3 | 22.7 | 6.99 | 1.193 | 1.293667 | 2.886333 |
| acyl carrier protein | 52 | 2 | 15.6 | 3.89 | 1.1645 | 1.0205 | 1.719 |
| pyruvate dehydrogenase E1 component subunit beta | 51 | 2 | 6.4 | 4.6 | 0.625 | 1.218 | 2.308 |
| deoC/LacD family aldolase family protein | 211 | 5 | 18.8 | 6.39 | 1.036667 | 1.001667 | 2.615667 |
| cyclophilin type peptidyl-prolyl cis-trans isomerase/CLD family protein | 21 | 3 | 13.5 | 5.48 | 0.9615 | 1.081 | 2.8395 |
| dihydrolipoyllysine-residue succinyltransferase, E2 component of oxoglutarate dehydrogenase (succinyl-transferring) complex | 55 | 2 | 3 | 5.88 | 1.142 | 1.077 | 2.857333 |
| oxoglutarate dehydrogenase (succinyl-transferring), E1 component | 181 | 8 | 8.2 | 5.81 | 1.112667 | 1.055 | 3.108333 |
| glyoxalase/Bleomycin resistance protein/Dioxygenase superfamily protein | 28 | 2 | 13.4 | 5.42 | 0.844 | 1.056 | 1.79 |
| peptidase family M20/M25/M40 family protein | 24 | 2 | 4.6 | 4.8 | 1.283667 | 1.35 | 2.879667 |
| succinyl-CoA synthetase beta chain (SCS-beta) | 55 | 3 | 7.7 | 5.3 | 1.388333 | 1.27 | 3.535333 |
| malate dehydrogenase, NAD-dependent | 272 | 5 | 13.8 | 5.07 | 1.088667 | 1.099333 | 2.461333 |
| succinate-semialdehyde dehydrogenase [NADP+] (SSDH) | 65 | 6 | 10.7 | 5.26 | 1.184 | 1.054333 | 2.962667 |
| succinate dehydrogenase iron-sulfur subunit | 26 | 2 | 8.9 | 7.93 | 1.076 | 1.206 | 3.163 |
| nitroreductase family protein | 21 | 2 | 12.6 | 5.21 | 0.816 | 1.043 | 2.346 |
| superoxide dismutase [Fe] | 436 | 3 | 16.6 | 5.21 | 1.065 | 1.024667 | 1.765 |
| sorbose/sorbosone dehydrogenase | 28 | 4 | 11.3 | 9.57 | 1.048 | 0.84 | 2.994 |
| preprotein translocase, YajC subunit | 78 | 2 | 14.9 | 9.63 | 1.125 | 1.465 | 1.987 |
| protein-export membrane protein SecF | 25 | 2 | 2.2 | 4.85 | 0.868 | 0.511 | 2.101 |
| aconitate hydratase 1 | 24 | 4 | 3.5 | 5.02 | 0.902 | 1.45 | 3.326 |
| integration host factor, alpha subunit | 24 | 2 | 10.1 | 9.97 | 0.861 | 0.932 | 1.652 |
| ribosomal protein L32 | 21 | 2 | 29.6 | 5.33 | 0.419 | 1.27 | 1.102 |
| orotate phosphoribosyltransferase (OPRT) (OPRTase) | 46 | 2 | 6.3 | 5.88 | 1.197 | 1.187 | 1.674 |
| ATP-dependent protease La | 34 | 3 | 2.5 | 5.54 | 1.2595 | 1.24 | 1.9735 |
| ribosomal protein S2 | 141 | 5 | 15.5 | 5.11 | 1.058667 | 1.259333 | 3.502667 |
| translation elongation factor Ts | 72 | 4 | 10.6 | 4.91 | 1.034667 | 1.131667 | 2.89 |
| ABC transporter, periplasmic substrate-binding protein | 54 | 3 | 9.3 | 4.5 | 1.037333 | 1.006333 | 1.584333 |
| uncharacterized protein family (UPF0051) family protein | 20 | 3 | 6.1 | 4.84 | 0.965 | 1.333 | 1.55 |
| feS assembly ATPase SufC | 40 | 5 | 16.8 | 5.06 | 1.019 | 1.379 | 1.852 |
| GTP-binding protein TypA/BipA | 20 | 3 | 3.3 | 5.12 | 1.2665 | 1.325 | 2.9165 |
| alanyl-tRNA synthetase | 39 | 7 | 6.7 | 5.3 | 1.0725 | 1.15 | 2.0095 |
| protein RecA | 111 | 4 | 10.8 | 5.28 | 1.069333 | 1.276333 | 2.218 |
| bacterial extracellular solute-binding proteins, family 5 Middle family protein | 42 | 2 | 5 | 4.41 | 1.0315 | 0.943 | 1.575 |
| ABC transporter family protein | 21 | 2 | 4.7 | 9.82 | 0.8985 | 1.037 | 1.782 |
| bacterial extracellular solute-binding proteins, family 5 Middle family protein | 19 | 2 | 1.9 | 4.6 | 0.961 | 0.897 | 1.477 |
| uncharacterized peroxidase-related enzyme family protein | 40 | 3 | 16.2 | 4.63 | 1.1725 | 1.486 | 2.4795 |
| bacterial extracellular solute-binding proteins, family 5 Middle family protein | 26 | 3 | 6 | 4.65 | 1.076 | 1.232 | 2.035 |
| NLPA lipofamily protein | 573 | 8 | 23.3 | 4.22 | 1.167333 | 1.154333 | 1.804333 |
| bacterial extracellular solute-binding family protein | 119 | 5 | 16.9 | 4.08 | 1.126667 | 1.176333 | 1.963667 |
| glycine cleavage system H protein | 50 | 2 | 7.6 | 3.87 | 0.984 | 1.063 | 1.776 |
| 6-phosphogluconolactonase | 25 | 2 | 4 | 5.13 | 1.19 | 1.085 | 2.142 |
| nucleoside diphosphate kinase family protein | 36 | 2 | 10.2 | 4.32 | 0.89 | 1.607 | 2.776 |
| cytosol aminopeptidase family, catalytic domain protein | 19 | 3 | 4.7 | 5.97 | 0.64 | 0.969 | 2.023 |
| ribosomal protein S9/S16 family protein | 96 | 3 | 17.9 | 10.85 | 1.260333 | 1.131333 | 3.219 |
| ribosomal protein L13 | 144 | 4 | 26.8 | 10.04 | 1.164333 | 1.310333 | 2.851 |
| NAD | 38 | 2 | 4.6 | 5.67 | 1.053 | 1.148 | 2.522333 |
| NAD(P)(+) transhydrogenase (AB-specific), alpha subunit | 41 | 2 | 3 | 5.8 | 0.776 | 0.9555 | 1.552 |
| cobaltochelatase, CobS subunit | 29 | 3 | 9.6 | 5.66 | 0.93 | 1.17 | 1.667667 |
| NADPH-dependent FMN reductase family protein | 38 | 3 | 17.7 | 5.09 | 1.115333 | 1.219 | 2.338 |
| acetyl-CoA carboxylase, biotin carboxyl carrier protein | 21 | 2 | 4.3 | 4.79 | 1.351 | 1.563 | 4.127 |
| acetyl-CoA carboxylase, biotin carboxylase | 22 | 3 | 6.5 | 5.6 | 1.556 | 1.372 | 2.4775 |
| ATP-dependent Clp protease, ATP-binding subunit ClpX | 28 | 4 | 9.2 | 5.32 | 0.88 | 0.908 | 2.808 |
| clp protease family protein | 265 | 5 | 24.2 | 5.5 | 1.066 | 1.075333 | 1.86 |
| conserved hypothetical protein | 936 | 4 | 41 | 5.88 | 1.012667 | 1.717667 | 1.522333 |
| transaldolase, putative | 135 | 7 | 30 | 4.75 | 1.043 | 0.987333 | 1.698 |
| DNA topoisomerase I | 29 | 5 | 4.8 | 8.18 | 0.7905 | 1.198 | 2.7965 |
| histidine kinase-, DNA gyrase B-, and HSP90-like ATPase family protein | 22 | 2 | 2.4 | 5.06 | 0.934 | 1.08 | 2.648 |
| peptidase T | 65 | 4 | 9.2 | 4.82 | 0.983333 | 0.812333 | 2.071333 |
| flavin reductase like domain protein | 18 | 2 | 9.6 | 6.38 | 1.0255 | 0.9145 | 1.442 |
| glucans biosynthesis protein G | 23 | 2 | 4.4 | 4.98 | 1.071 | 1.367 | 2.166 |
| sorbose/sorbosone dehydrogenase | 327 | 4 | 8.6 | 4.35 | 1.062 | 1.105 | 1.41 |
| conserved hypothetical protein | 34 | 2 | 10.1 | 4.95 | 1.232667 | 1.443667 | 3.929333 |
| oppF | 20 | 2 | 4.8 | 9.06 | 1.131667 | 1.221333 | 2.635 |
| bacterial extracellular solute-binding proteins, family 5 Middle family protein | 26 | 2 | 4.4 | 4.22 | 1.115 | 0.832 | 1.482 |
| H-NS histone family protein | 20 | 4 | 40 | 8.96 | 0.969667 | 0.801333 | 2.293667 |
| ribosomal protein S21 | 20 | 2 | 25 | 11.27 | 1.282 | 0.591 | 1.755 |
| N-6 DNA Methylase family protein | 21 | 2 | 1.8 | 5.12 | 1.252 | 1.372 | 2.395 |
| ribosomal protein L27 | 29 | 2 | 13.5 | 11.03 | 1.105 | 0.875 | 1.526 |
| ribosomal protein L21 | 57 | 2 | 17.8 | 10.19 | 0.9985 | 1.126 | 1.788 |
| large conductance mechanosensitive channel protein | 80 | 3 | 19.6 | 7.98 | 0.927 | 0.873333 | 2.077333 |
| conserved hypothetical protein | 153 | 4 | 25.3 | 6.51 | 1.039667 | 1.088667 | 1.819667 |
| phenylalanyl-tRNA synthetase, beta subunit | 24 | 5 | 5.1 | 5.16 | 1.367 | 1.041 | 1.916 |
| pyruvate carboxylase | 22 | 6 | 4.5 | 5.76 | 1.045667 | 1.172 | 2.185667 |
| phenylalanyl-tRNA synthetase, alpha subunit | 27 | 4 | 8.4 | 5.26 | 0.644 | 0.976 | 1.421 |
| conserved hypothetical protein | 25 | 2 | 3.8 | 4.77 | 1.057 | 0.854 | 1.565 |
| ribosomal protein L20 | 31 | 2 | 8.3 | 10.99 | 1.299333 | 1.614 | 2.527667 |
| hsp20/alpha crystallin family protein | 318 | 5 | 36.2 | 6.29 | 1.158 | 1.225 | 0.998667 |
| phosphate ABC transporter, ATP-binding protein | 27 | 3 | 13.8 | 5.6 | 2.245 | 1.84 | 3.398 |
| hemolysin-type calcium-binding region | 24 | 2 | 2.1 | 5.33 | 1.39 | 0.796 | 0.432 |
| aminotransferase class IV family protein | 32 | 2 | 3.5 | 5.18 | 1.119333 | 1.210333 | 1.778333 |
| translation initiation factor IF-1 | 18 | 2 | 18.1 | 9.52 | 0.975 | 0.932 | 1.851333 |
| carboxynorspermidine decarboxylase | 18 | 4 | 7.1 | 5.01 | 1.268 | 1.5 | 1.999 |
| phosphopyruvate hydratase | 330 | 7 | 18.2 | 4.66 | 1.159333 | 1.202333 | 3.065333 |
| N-acetyl-gamma-glutamyl-phosphate reductase | 100 | 6 | 17 | 5.54 | 1.133333 | 1.145667 | 1.825333 |
| phosphoribosylformylglycinamidine synthase II | 25 | 2 | 1.8 | 4.69 | 1.148 | 1.096 | 1.8325 |
| transketolase | 90 | 4 | 7.1 | 7.9 | 0.934333 | 0.963667 | 1.743667 |
| glyceraldehyde-3-phosphate dehydrogenase, type I | 589 | 5 | 14.1 | 5.57 | 1.205333 | 1.377333 | 3.384 |
| tellurite resistance protein | 41 | 7 | 19 | 4.97 | 0.863333 | 0.902 | 1.91 |
| protein TolR | 26 | 2 | 4.5 | 4.9 | 1.046667 | 1.622 | 5.555 |
| calcineurin-like phosphoesterase family protein | 27 | 3 | 5.7 | 4.18 | 1.0635 | 0.9465 | 2.882 |
| serine hydroxymethyltransferase (Serine methylase)(SHMT) | 28 | 4 | 6.8 | 5.79 | 1.104333 | 0.964667 | 2.057667 |
| ribosomal protein L28 | 85 | 2 | 19.4 | 10.27 | 1.384667 | 1.323333 | 2.557333 |
| csbD-like family protein | 22 | 3 | 36.9 | 4.73 | 1.0055 | 1.1515 | 2.025 |
| glutathione S-transferase, C-terminal domain protein | 25 | 2 | 2.8 | 5.95 | 0.983 | 1.367 | 1.885 |
| NADPH-dependent FMN reductase family protein | 19 | 2 | 3.3 | 5.54 | 1.175 | 1.796 | 2.138 |
| nitrogen regulatory protein P-II (PII signal transducing protein) | 20 | 3 | 27.7 | 5.21 | 1.535 | 1.181 | 2.689 |
| glutamine synthetase, type I | 901 | 10 | 19 | 4.99 | 1.143667 | 1.236 | 2.735 |
| asparate kinase, monofunctional class | 68 | 6 | 11.9 | 5.04 | 0.973333 | 1.046333 | 1.803667 |
| catalase/peroxidase HPI | 41 | 5 | 6.8 | 4.81 | 0.9685 | 1.7705 | 2.7185 |
| metE | 43 | 3 | 8.5 | 5.34 | 0.932333 | 1.128667 | 2.103667 |
| bacterial extracellular solute-binding proteins, family 5 Middle family protein | 27 | 2 | 2.4 | 4.65 | 2.3435 | 2.4275 | 6.525 |
| imidazoleglycerol-phosphate dehydratase (IGPD) | 20 | 2 | 9.7 | 5.83 | 1.135 | 1.118 | 2.664 |
| conserved hypothetical protein | 58 | 5 | 16.1 | 4.01 | 0.953 | 0.991333 | 1.982667 |
| insulinase (Peptidase family M16) family protein | 36 | 2 | 2.6 | 4.59 | 1.163 | 1.108 | 1.8435 |
| ahpC/TSA family protein | 735 | 11 | 51.6 | 5.16 | 1.035 | 1.019 | 2.130333 |
