# Supplementary figures and images for: Proteomic Analysis of Ketogulonicigenium vulgare under Glutathione Reveals High Demand for Thiamin Transport and Antioxidant Protection
Source: PLoS One. 2012 Feb 22;7(2):e32156. doi: 10.1371/journal.pone.0032156 (PMC3284542; doi:10.1371/journal.pone.0032156)

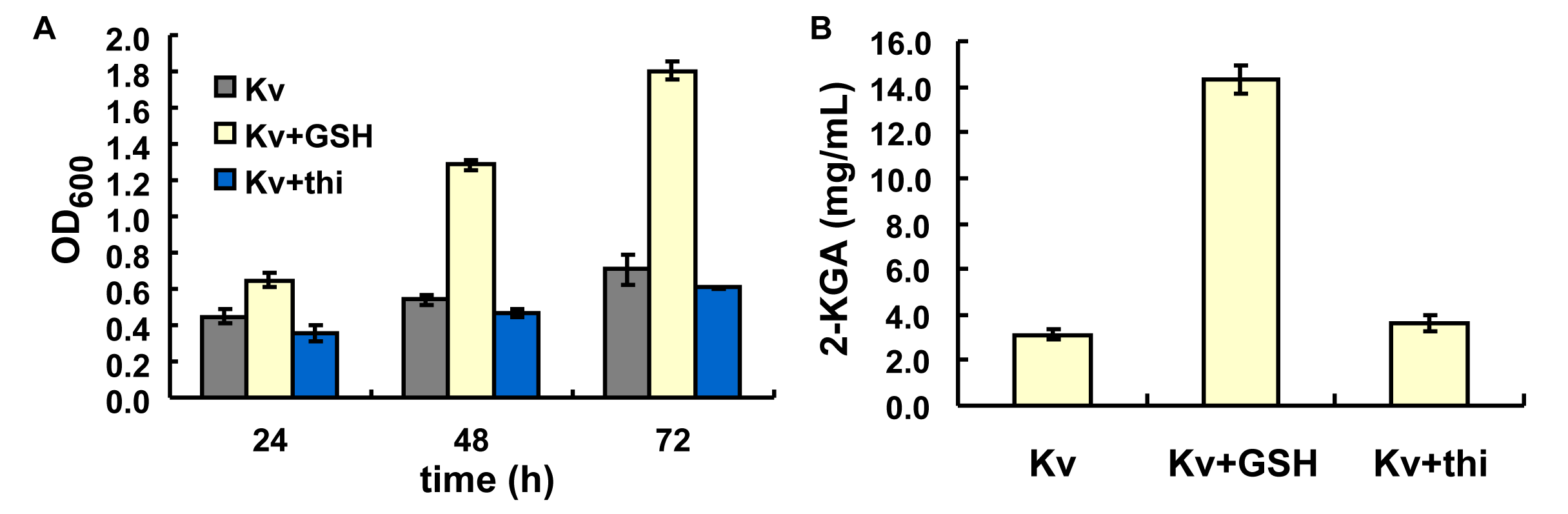

Supplement: Figure S1 — Growth and 2-KGA production of K. vulgare after adding thiamin: (A) Optical density of K. vulgare after adding thiamin compared with control grown with and without GSH; (B) 2-KGA production of K. vulgare at 72 h after adding thiamin compared with control grown with and without GSH. (TIF) [file pone.0032156.s002.tif]
